# Interventions to prevent violence against women and girls globally: a global systematic review of reviews to update the RESPECT women framework

**DOI:** 10.1136/bmjph-2024-001126

**Published:** 2025-01-20

**Authors:** Chelsea Ullman, Avni Amin, Angela Bourassa, Shikha Chandarana, Flávia Dutra, Mary Ellsberg

**Affiliations:** 1 Global Women's Institute, The George Washington University, Washington, Washington, USA; 2 Sexual and Reproductive Health and Research, World Health Organization, Geneva, Switzerland; 3 The George Washington University, Washington, District of Columbia, USA

**Keywords:** Public Health, Sex Factors, Female, Public Health Practice, Primary Prevention

## Abstract

**Objective:**

The field of violence against women and girls (VAWG) prevention research has rapidly advanced, with ample evidence now demonstrating that VAWG is preventable. The aim of this systematic review of reviews is to update the evidence that underpins the RESPECT women framework with the newest available evidence from 2013 onward.

**Methods:**

Academic and non-academic databases were searched using terms related to VAWG prevention (January 2013–April 2022). Evaluations had to have a target population of women or girls aged 10 and older. Data were extracted from included reviews, and AMSTAR-2 was used to assess the risk of bias in systematic reviews. The primary outcome of interest was change in any form of VAWG, including physical, sexual and emotional intimate partner violence and sexual violence and harassment from non-partners, including child and adolescent sexual abuse.

**Results:**

From the thousands of articles screened, 178 were included in this study. Six (3%) reviews focused on interventions that aim to strengthen relationship skills, 14 (8%) focused on empowerment of women and girls, 79 (44%) on services for survivors, 5 (3%) on poverty reduction, 16 (9%) on creating safe environments, 36 (20%) on preventing child and adolescent abuse and 22 (12%) on transforming gender attitudes, beliefs and norms. Little new evidence has emerged to meaningfully change the nature and strength of evidence for interventions related to relationship skills strengthening and poverty reduction. However, there is new evidence to reflect the effectiveness of select types of interventions across settings in the other five strategies.

**Conclusion:**

Despite progress in VAWG prevention research, significant gaps in the evidence base persist. Further research is needed to explore intervention areas and marginalised populations in various contexts. Several programmatic approaches exhibiting efficacy in low-income and middle-income countries remain unexplored and unevaluated in high-income countries, and vice versa, warranting further adaptation and evaluation.

**WHAT IS ALREADY KNOWN ON THIS TOPIC**

**WHAT THIS STUDY ADDS**

- Substantial evidence has been generated since 2013, and this systematic review of reviews updates the global evidence base underpinning prevention of VAWG.

**HOW THIS STUDY MIGHT AFFECT RESEARCH, PRACTICE OR POLICY**

- This systematic review of reviews was conducted with the express purpose of updating the evidence underpinning the UN interagency RESPECT women framework, which serves as global guidance for VAWG policy-makers and practitioners.

## Introduction

 Violence against women and girls (VAWG) is a global public health problem and a violation of human rights. Globally, 27% of ever-partnered women 15–49 years of age have experienced physical and/or sexual violence by a current or former intimate partner at least once in their lifetime.^1^ This figure is likely an underestimate because (1) VAWG is stigmatised and hence may be under-reported and (2) this figure excludes many other forms of violence, including psychological intimate partner violence (IPV), sexual harassment in schools, public spaces and workplaces, intimate partner homicide, forced marriages and trafficking.^2 3^ Prevalence of IPV in the past 12 months is much lower in World Bank high-income countries (HICs) compared with low-income and middle-income countries (LMICs). Women living with disabilities, indigenous women and transwomen also have higher rates of violence compared with women in the general population.^4^

All forms of VAWG are rooted in gender inequalities that perpetuate unequal gender norms, promoting male privilege and power, women’s subordination and the acceptance of violence. These inequalities are reinforced through economic, legal and cultural aspects of women’s and girls’ lives, and interventions that address these complex dimensions are needed to root out VAWG.

### Preventing violence and the RESPECT women framework

There is now ample evidence that VAWG is preventable,^5^ and researchers are building knowledge about what programmes work to prevent violence. Decades of innovations in programming, activism by feminist organisations and investments in rigorous evaluations of interventions have contributed to a growing body of promising approaches.

To synthesise the growing evidence of what works to prevent VAWG, in 2019, the WHO and UN Women (in conjunction with 12 other multilateral and bilateral agencies) published the RESPECT women framework, which aims to advance and scale up evidence-based prevention programming. RESPECT has become a useful tool for policy-makers, practitioners and advocates in the VAWG prevention field.^6^

The RESPECT framework was based on evidence from a previous systematic review of reviews^3 7^ focused on VAWG prevention interventions, as well as several additional systematic reviews of specific prevention strategies. The strategies included in the RESPECT women framework reflect approaches across levels of the socioecological model (eg, individual, relationship, community and society), acknowledging that work at each of these levels is required to end VAWG. These approaches are summarised within the RESPECT women framework in table 1.

**Table 1:** RESPECT women framework

	Definition	Types of interventions included
R	Relationship skills strengthened	Group-based workshops with women and men to promote egalitarian attitudes and relationships. Couples counselling and therapy.
E	Empowerment of women	Empowerment training for women and girls including life skills, safe spaces, mentoring. Inheritance and asset ownership policies and interventions. Microfinance or savings and loan programmes that include gender and empowerment training components.
S	Services ensured	Empowerment counselling interventions or psychological support to support access to services (ie, advocacy). Alcohol misuse prevention interventions. Shelters/safe accommodation. Hotlines. One-stop crisis centres. Perpetrator interventions. Women’s police stations/units. Screening in health services. Sensitisation and training of institutional personnel without changing the institutional environment.
P	Poverty reduced	Economic transfers, including conditional/unconditional cash transfers plus vouchers and in-kind transfers. Labour force interventions including employment policies, livelihood and employment training. Microfinance or savings interventions without any additional components.
E	Environments made safe	Infrastructure and transport. Bystander interventions. Whole-school interventions.
C	Child and adolescent abuse prevented	Home visitation and health worker outreach. Parenting interventions. Psychological support interventions for children who experience violence and who witness intimate partner violence. Life skills/school-based curriculum, rape and dating violence prevention training.
T	Transformed attitudes, beliefs and norms	Community mobilisation. Group-based workshops with women and men to promote changes in attitudes and norms. Social marketing or edutainment and group education. Group education with men and boys to change attitudes and norms. Stand-alone awareness campaigns/single component communications campaigns.

The evidence was organised to develop seven strategies, with each letter of ‘RESPECT’ representing one strategy to prevent or reduce VAWG. More information about each of the seven RESPECT Framework strategies is included in online supplemental appendix 1.

There has been a proliferation of evidence on what works to prevent VAWG over the past 5–10 years emerging from several large-scale initiatives supporting prevention programming and evaluations. These initiatives include the What Works to Prevent Violence against Women programme, funded by the UK Government, and the Spotlight initiative, funded by the European Union and administered by the UN.^3 5^ Therefore, a systematic review of reviews is needed to synthesise this extensive new evidence, update the global knowledge base and revise the RESPECT women framework to reflect this progress. The present systematic review of reviews aims to update the underlying evidence on what works to prevent VAWG, which is the evidence that informs the RESPECT women framework.

## Methods

A systematic review of reviews (as opposed to a systematic review) was selected methodologically in this case due to this method’s strength in synthesising broader trends in the literature in a field where evidence has rapidly proliferated (as in this case with VAWG prevention over this time period). Each of the seven strategies of the RESPECT women framework is underpinned by multiple interventions and a number of systematic reviews that answer specific questions about the bodies of evidence. A systematic review of reviews was deemed more appropriate in this instance to identify bodies of evidence that underpin the seven strategies more widely, rather than to answer specific effectiveness questions for the interventions in each strategy. Because we were seeking to update the seven broad strategies in the RESPECT framework, this method was more appropriate, rather than a systematic review which could have addressed a narrower research question.

This systematic review of reviews takes as a starting point the protocol for a previous systematic review of reviews^7^ in 2013 that was one of the main sources of evidence in the development of the RESPECT women framework. The protocol of the present research adheres to the Preferred Reporting Items for Systematic Reviews and Meta-Analyses (PRISMA)^8^ guidelines. The protocol for this review is registered with PROSPERO (2022 CRD42022315919).

### Research questions

The research questions guiding this review of reviews were as follows. At the global level:

- What types of interventions are effective in preventing and/or reducing different forms of violence against women with a focus on IPV including dating violence, non-partner sexual violence (NPSV) including sexual harassment and child sexual abuse?
- What intervention characteristics make interventions to prevent violence against women and girls successful?
- What is the quality of the available evidence, including strength and magnitude of effect sizes?

### Inclusion and exclusion criteria

We included both systematic and comprehensive reviews presenting empirical results from at least two experimental or quasi-experimental intervention studies aiming to reduce or prevent VAWG. Comparison groups were those that received no intervention or received the standard of care, or another intervention. Included study designs were either randomised or not, appropriate to the type of intervention. Reviews that only described interventions or processes without evaluating them, reviews of intervention evaluations which lacked a control or comparison group (pre–post studies), and qualitative reviews were all excluded from this review of reviews.

Included reviews contained intervention evaluations that had a target population of women or girls aged 10 and older, including transwomen, who are subject to violence, or men and boys aged 10 and older, including transmen, who are perpetrators (or potential perpetrators) of VAWG. This age limit allowed us to focus on the gendered violence affecting adolescent girls, as well as women. There is clear evidence that gendered differences in social norms, behaviours and attitudes that underpin violence against women often emerge at puberty, and that these norms and attitudes form early in the lives of adolescents.^9^ Therefore, it was important to pick this age group as the lower limit.

Interventions must evaluate either (1) change in incidence or prevalence of one or more types of VAWG, such as physical, sexual or emotional IPV, sexual violence and harassment from non-partners or child and adolescent sexual abuse (primary outcomes) or (2) change in behaviours, attitudes and social norms that regulate the acceptability of VAWG. These can include, for example, attitudes that condone VAWG in general or under specific circumstances, gender equitable attitudes and norms, attitudes towards intervening in VAWG, changes in women’s and girls’ decision-making, and power imbalances within intimate relationships (secondary outcomes). A review was included if it included either the primary or the secondary outcome of interest (several reviews addressed both outcomes).

Forms of violence against children such as bullying, neglect and youth violence as well as harmful practices, such as female genital mutilation or child marriage, were excluded from this review due to how substantively they are addressed through other bodies of evidence, including those that underpin the UN interagency INSPIRE framework to prevent violence against children.^10^ Reviews of interventions focusing exclusively on outcomes other than these (eg, increased screening for IPV in health settings, which did not report changes in violence or norms as outcomes) were excluded.

Reviews must have been published in English, Spanish or French between May 2013 and April 2022 and may be peer-reviewed or grey literature. Book sections and chapters and PhD theses were excluded.

### Search strategy

The search strategy combined terms related to reductions in VAWG and changes in gender attitudes, beliefs and norms, review types and intervention types relevant to this review. The researchers’ search terms were developed based on the current evidence base related to VAWG prevention and were refined in consultation with librarians from George Washington University’s Himmelfarb Library. The present review relied on an updated search strategy from the 2013 systematic review of review.^3^ A full list of search terms can be found in online supplemental appendix 2.

We conducted searches of relevant literature sources in April 2022, capturing potential reviews for inclusion from electronic databases of peer-reviewed literature and grey literature. Databases used for peer-reviewed literature include PsycINFO, PubMed, JSTOR, SCOPUS, EMBASE, MEDLINE, ASSIA, Social Services Abstracts, CINAHL Plus with Full Text, ERIC, EconLit and the Cochrane Database of Systematic Reviews, with results filtered by publication date and language. Spanish language literature was also searched through the following databases using a Spanish translation of the search terms: LILACS, IBECS and SciELO database. No additional articles were derived from these sources. Grey literature was searched from the organisational websites (eg, WHO), registries (eg, PROSPERO) and academic search engines (eg, Google Scholar). Additional literature was solicited from experts in VAWG research and programming based on a list of experts and colleagues developed by the authors and sourced from bibliographies of prior systematic reviews and reviews of reviews captured by our search terms.

Search results from all databases and registries were compiled and imported into Covidence, where duplicates were then eliminated. Title and abstract screening were conducted by three independent reviewers (CU, AB and SC), with conflicts resolved through discussion and consensus. Full-text screening was conducted following the same process.

### Data extraction and risk of bias

Data were extracted by two independent reviewers using a shared extraction form. While meta-analyses were included, their findings were included in a primarily qualitative way so as to be compiled and assessed alongside findings from other reviews. All extracted reviews were uploaded to Eppi-reviewer for further coding with the AMSTAR-2 Risk of Bias tool. Systematic reviews and meta-analyses were coded independently (by CU, AB, SC, and FD) and in duplicate using the AMSTAR-2^11^ code set. AMSTAR is a critical appraisal tool for systematic reviews and meta-analyses of randomised health trials. AMSTAR-2 is an assessment tool with 16 dimensions that expands on AMSTAR to include systematic reviews and meta-analyses of non-randomised studies. Non-systematic reviews, such as literature reviews, narrative reviews and other reviews of evidence included in this review, were not assessed due to inappropriate fit with the AMSTAR-2 tool, though the evidence from these reviews was included in the present analysis. AMSTAR-2 ratings are explained in table 2.

**Table 2:** AMSTAR-2 ratings

High	Review includes no or one non-critical weakness
Moderate	Review includes more than one non-critical weakness
Low	One critical flaw with or without non-critical weaknesses
Critically low	More than one critical flaw with or without non-critical weaknesses

AMSTAR, A MeaSurement Tool to Assess systematic Reviews.

While all included reviews were assessed for risk of bias using the AMSTAR-2 tool detailed above, individual evaluations included in each review were not assessed for risk of bias, given the focus here on review of reviews.

### Analysis strategy

In the RESPECT women framework, each prevention approach across the seven strategies was assessed using the following rating system in both LMIC and HIC settings. If a strategy had:

- >1 evaluation showing a significant reduction in violence outcomes, it was categorised as promising.
- >1 evaluation showing significant improvements in intermediate outcomes related to violence (ie, changes in norms or in women’s agency or power in relationships), it was categorised as more evidence needed.
- Evaluations showing conflicting results in reducing violence, it was categorised as conflicting.
- No interventions that had yet been rigorously evaluated, it was categorised as no evidence.
- >1 evaluation showing no reductions in violence outcomes, it was categorised as ineffective.

For consistency, this same assessment tool was used in the analysis of the articles included in the present systematic review of reviews. The researchers compared the findings from the present research to the original assessments in the 2019 RESPECT women framework and changed assessments based on the above system as needed. Further, because this was a systematic review of reviews rather than a systematic review, analysis of the findings is only at the level of the review, rather than into the detail of each individual evaluation. While we extracted data at both the review level and the individual evaluation level to inform update of the RESPECT women framework, here we have only reported the findings at the review of reviews level.

Because this systematic review of reviews was inclusive of both meta-analyses and systematic reviews that did not pool statistical results, overall findings from each article were assessed on a case-by-case basis and not using a single effect measure.

### Findings

**Figure 1:**
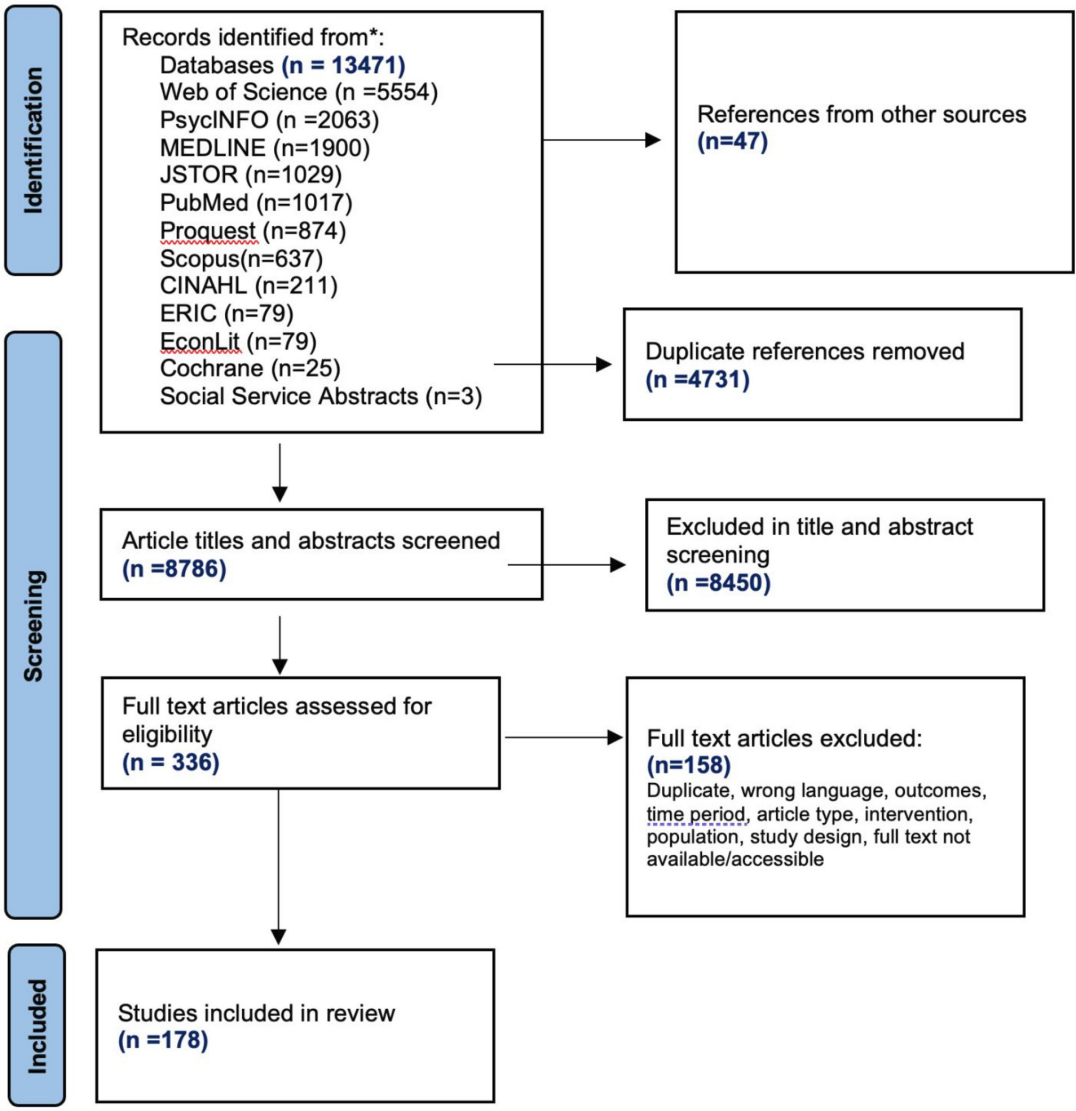
PRISMA flow chart. *Page *et al*.^8^ PRISMA, Preferred Reporting Items for Systematic Reviews and Meta-Analyses.

The database searches initially yielded 13 518 references, which were imported into Covidence. Additional articles from hand searches and grey literature searches were added. After removing duplicates and completing title and abstract screening, 336 articles were eligible for full-text screening. An additional 158 were excluded during full-text screening (reasons included either duplication or wrong language, outcomes, time period, article type, intervention, population or study design) and the remaining 178 reviews are included in the current review (see figure 1).

More detailed information about each systematic review categorised by each of the seven RESPECT strategies can be found in online supplemental appendix 3, and a full list of citations for the 178 included reviews can be found in online supplemental appendix 4. Two-thirds (118 out of 178) of the included articles were systematic reviews, and one-fifth (37/178) included meta-analysis. The remainder were categorised as scoping, rapid or literature reviews based on their methodology, or comprehensive, evidence, or narrative reviews based on their structure. Nearly all reviews (165 or 92% of all included articles) reported directly on the primary outcome—violence reduction—and the majority (109 or 62%) either reported only on secondary outcomes, or on secondary outcomes in addition to primary outcomes—changes in norms, attitudes, beliefs. Most of the 94 reviews included both primary and secondary outcomes; in other words, they measured both changes in violence and changes in attitudes, norms and beliefs, while 70 reviews included only the primary outcome, and 14 reviews included only the secondary outcome.

Distribution of included reviews across seven RESPECT strategies was inconsistent, with a majority of reviews continuing to focus on interventions related to services (ie, the ‘S’ strategy). Only six (3%) reviews focused on interventions that aim to strengthen relationships (R), 14 (8%) focused on empowerment of women and girls (E), 79 (45%) on services ensured (S), 5 (3%) on poverty reduction (P), 16 (9%) on environments made safe (E), 36 (20%) on child and adolescent abuse prevention (C) and 22 (12%) on transformed attitudes, beliefs and norms (T). This is an approximate breakdown, however, as some reviews included interventions that spanned several of the RESPECT strategies. Overall, just over half of the reviews (94, about 53%) included studies from LMICs, and within these reviews, about a quarter of the individual intervention studies that were included were from LMICs.

**Figure 2:**
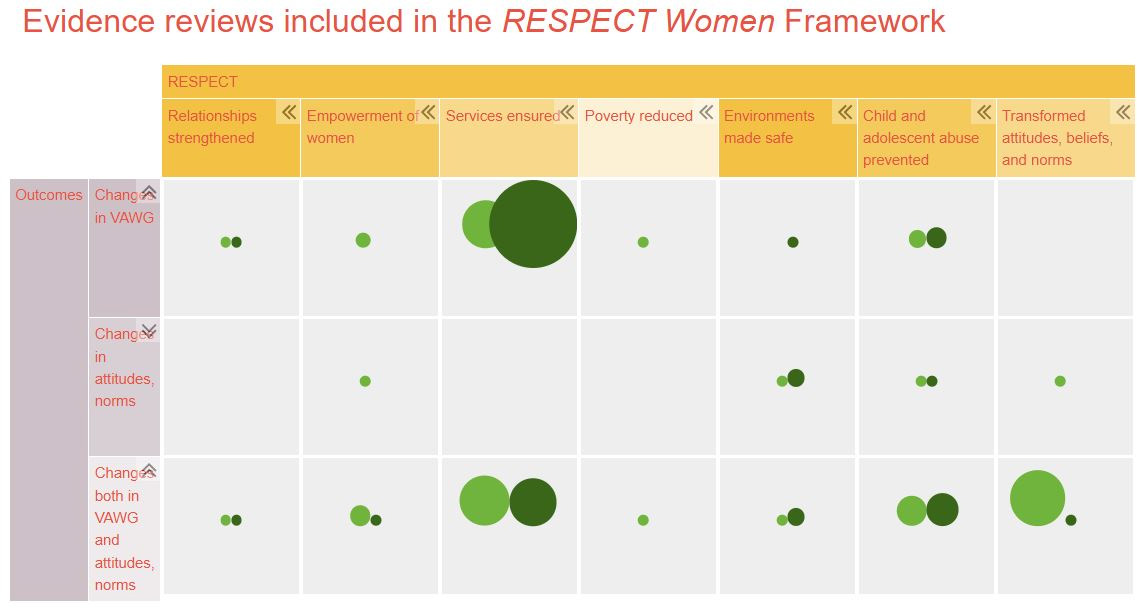
Evidence and gap map. VAWG, violence against women and girls.

We generated an interactive evidence and gap map (https://respect-prevent-vaw.org/gap-map) to summarise the reviews found across each strategy of RESPECT, whether these reviews include interventions in LMICs or not, and what outcomes were included across reviews (see figure 2). Light green circles indicate evidence from LMICs, and dark green circles indicate evidence from HICs. The size of the circle indicates the amount of evidence generated in that strategy, across the primary and secondary outcomes.

Summary findings of the reviews from each of the seven categories are detailed below.

### Relationship skills strengthened

Only six reviews included interventions in the relationship skills strengthened strategy. All six showed reductions in VAWG as an outcome, and four of the six also included changes in norms that support VAWG as an outcome. The body of evidence in this strategy is mixed across HICs and LMICs (three reviews in this strategy included LMICs, the other three did not). Most (five of six) of these reviews were rated critically low on the AMSTAR-2 rating.

The relationship skills strengthened category primarily includes two programmatic approaches: (1) group-based workshops with women and men to promote egalitarian attitudes and relationships and (2) couples counselling and therapy. When the RESPECT women framework was originally published in 2019, the evidence base showed the promising effectiveness of group-based workshops with women and men in LMICs, but more evidence was needed on these approaches in HICs. Conversely, at the time, couples counselling and couples therapy had demonstrated promising effectiveness in HICs, but more evidence was needed in LMICs. Four of the six new reviews included in the present study were entirely focused on couples counselling, involving intervention with all couples regardless of whether or not there was abuse. Couples therapy involving relationships with a pre-established pattern of abuse were not considered in the systematic reviews of interventions from LMICs. These new reviews did not reveal sufficient new evidence of effectiveness to change the original assessment that more evidence is needed to demonstrate effectiveness in LMICs.

More evidence is still needed in LMICs to explore the effectiveness of couples counselling and couples therapy as two distinct types of interventions, with the former focused on all couples, and the latter on couples where there is already abuse. Concerns have also been flagged regarding the appropriate of couples therapy in all situations, particularly where IPV and/or coercive control is used to terrorise and exert power over the abused partner.^12^

### Empowerment of women

Fourteen reviews included interventions in the empowerment of women strategy. All but one of these reviews showed reductions in violence as an outcome, and most (9 of 14) showed changes in norms that support VAWG as an outcome. Nearly all (13 out of 14) of these reviews included individual evaluation studies from LMICs. While the quality of the reviews varied, a preponderance of reviews was assessed as moderate or high using the AMSTAR-2 rating, indicating a relatively strong body of evidence.

Specific programmatic approaches within this strategy focus on social, economic and psychological empowerment of women. Interventions provide women with tools to strengthen economic outcomes and increase their autonomous decision-making, self-esteem and self-efficacy. These programmes can include educational sessions for women in healthy relationships, financial services, life and employment skills, self-defence, and community building. Almost all the interventions in this group involve short term or continuing education. Microfinance programmes are also included in this strategy, but only if the microfinance programme is paired with gender and/or empowerment training components (microfinance programmes without the added gender/empowerment training component would be found in the poverty reduced strategy).

The effectiveness of empowerment interventions on their own to prevent IPV can vary widely based on the programmatic approach. A large number of evaluations included in these reviews showed only changes in outcomes related to women’s efficacy, which are important intermediate outcomes in the pathway to reducing violence. However, many of the individual evaluations in the included reviews did not measure the impact on IPV (including dating violence), so it is difficult to conclude several of the specific programmatic approaches to empowerment of women are effective in preventing VAWG, despite a relatively strong body of evidence at the review level.

When the RESPECT women framework was first published, there was conflicting evidence from specific systematic reviews from LMICs that microfinance or savings and loans programmes with gender and empowerment training components reduced VAWG. The update offered by the present research establishes that there is promising evidence^13 14^ (from systematic reviews of moderate quality) that this specific programmatic approach is effective in reducing VAWG.

Similarly, the RESPECT framework originally suggested that more evidence was needed to understand whether empowerment training for women and girls (including life skills, safe spaces and mentoring) is effective in reducing VAWG in HICs. The present review changes this categorisation for the empowerment training approach to ‘promising’ based on the new evidence^15^,^17^ in the included reviews from HICs (which ranged in quality from low to moderate). This means that the global evidence base indicates that empowerment training for women and girls is, in fact, effective in reducing VAWG in both HICs and LMICs.

Finally, the present review includes evaluations of programmatic approaches not previously included in the RESPECT women framework. These include safety planning and decision-making interventions as part of the empowerment of women.^18^,^20^ While evaluations of these interventions did not measure either the primary or secondary outcomes specified in the protocol for the present research, the growth in evaluation of these programmatic approaches points to a need to add a new category of interventions to the empowerment of women strategy in the RESPECT women framework. These evaluations appeared in six reviews, most of which were of high quality.

Remaining evidence gaps within this strategy include data from HICs on the effectiveness of inheritance and asset ownership policies/interventions, and the effectiveness of microfinance and/or savings and loans with a gender empowerment component on reducing VAWG.

Overall, the systematic review of reviews revealed a stronger evidence base to support empowerment interventions as effective in reducing VAWG, particularly in LMICs.

### Services ensured

Intervention reviews categorised as services ensured for survivors of VAWG are the largest body of evidence, as these reviews represent 45% (79/178) of all included articles. All 79 reviews included reductions in violence as an outcome, and about half (33 out of 79) also included changes in norms that support VAWG as an outcome. Just over half of these 79 reviews included evaluations in LMICs. The AMSTAR-2 rating of the 79 reviews included a mix of assessments on the scale (critically low, low, moderate and high), reflecting a large body of evidence of mixed quality in terms of risk of bias.

As part of the services ensured strategy (S), a variety of types of services/programmatic approaches for survivors are included. These are empowerment counselling interventions or psychological support to support access to services (ie, advocacy); alcohol misuse prevention interventions; shelters; hotlines; one-stop crisis centres; perpetrator interventions; women’s police stations/units; screening in health services and sensitisation and training of institutional personnel without changing the institutional environment (changing of institutional environments is included in the environments made safe (E) strategy of RESPECT).

Perpetrator interventions were the focus of the majority of the 79 reviews categorised as part of the services ensured strategy (nearly half had a focus on perpetrator interventions). The RESPECT women framework had initially assessed perpetrator interventions as demonstrating ‘conflicting evidence’ in HICs and as ‘needing more evidence’ in LMICs. This review did not generate sufficient evidence to change the previous assessment of the perpetrator interventions category, highlighting the continued misdirected investments in perpetrator interventions despite mounting evidence of their failure to demonstrate effectiveness in lowering VAWG in HIC or LMIC contexts. However, it became clear during data extraction that the perpetrator interventions delivered in health versus justice settings should be considered as separate categories and assessed for the strength of evidence separately, which was not the case in the original RESPECT framework. Several reviews included evaluations of justice interventions (like protection orders and arrests), which are different from interventions delivered to perpetrators in a health setting. In the present review, most included reviews of service interventions were focused on protection orders, and the findings across primary and secondary outcomes are mixed. In general, there were no or small effects of these orders on recidivism (ie, repeat perpetration), and effects varied by definition of reoffence (with studies using reports from women survivors generally indicating higher levels of continuing violence than those using measures based on rearrest or order violation).^21 22^ The majority of perpetrator interventions are evaluated in HICs as they rely on well-resourced justice and health infrastructure. Findings also indicate that protection and restraining orders are more successful among specific, often lower-risk groups (eg, victims whose partners are not stalking them), and among those survivors with more resources.

Remaining evidence gaps in this strategy include further evaluation of the impact of one-stop crisis centres and women’s police stations (particularly in HICs) and screening in health services on VAWG.

The present review changes the previous categorisation of RESPECT with respect to empowerment counselling or psychological support interventions to access services from ‘more evidence needed’ to ‘promising evidence’^23^,^26^ in LMICs, given that the included reviews from LMICs (just over half in the services ensured category) contained more than one evaluation that demonstrated significant reductions in violence.

### Poverty reduced

Poverty is a well-established risk factor for IPV, with bidirectional links (ie, women living in poverty are more likely to have higher exposure to conflict and community violence, and exposure to violence is more likely to drive women to poverty).^27^ Only five reviews maintained an explicit focus on reducing poverty. All five reviews included intervention evaluations that included reductions in violence as an outcome, and two of the five also included changes in social norms that underpin violence. All five evaluations included data from LMICs. One review was assessed with AMSTAR-2 as being of moderate quality, three were of low quality, and one was not given an AMSTAR-2 rating (given it was not a systematic review but rather a narrative review and could thus not be meaningfully assessed with this tool).

Programmatic approaches in this strategy differ from the empowerment of women strategy in that these interventions exclusively seek to reduce household poverty without a gender equality focus, or a component explicitly aimed at reducing violence. Poverty reduction interventions include economic transfers (including conditional/unconditional cash transfers plus vouchers and in-kind transfers), labour force interventions (including employment policies, livelihood and employment training) and microfinance or savings interventions without any additional components.

When the RESPECT women framework was published, economic transfers had been assessed as ‘promising’ in their ability to reduce VAWG in LMICs (though needed more evidence in HICs), labour force interventions had been assessed as ‘promising’ in HICs (though more evidence was needed in LMICs) and microfinance interventions without any additional gender equality components did not seem to be effective in LMICs, with no evidence from HICs.

With only five systematic reviews in this category, this review did not generate sufficient evidence to change these previous assessments. This means that the above evidence gaps remain (with a significant gap in evidence evaluating microfinance programmes in HICs). However, data extracted from the individual systematic reviews suggest that interventions implemented in certain population subgroups may be more effective than when implemented in other subgroups. For example, while cash transfers demonstrate small to no effect on IPV in most evaluations among adult women, cash transfer studies involving adolescent girls showed positive effects on delayed sexual debut, marriage and childbearing, which are risk factors for IPV in adolescent girls.^28^ Similarly, unconditional cash transfers have demonstrated decreased physical and emotional IPV and controlling behaviours, though only among polygamous households.^29^

While this review does not shed additional light on the overall body of evidence for poverty reduction strategies with respect to VAWG outcomes, it does highlight the importance of exploring how poverty reduction interventions may be beneficial in reducing VAWG in specific populations that are disproportionately at risk of, or impacted by, IPV.

### Environments made safe

While data show women and girls face the highest risk of violence in their homes and from people they know and trust, they also face violence outside the home—be it in public spaces, at school or in the workplace. This violence restricts women’s ability to participate in public and civic life. The RESPECT women framework has outlined three areas in the environments made safe category (E) that address VAWG in contexts outside the home: interventions addressing infrastructure and transport, bystander interventions and whole-of-school approaches. 16 reviews (about 9%) in this review addressed interventions to make environments safe. Half of these reviews (8 of 16) used reductions in violence as an outcome, while nearly all of them used changes in norms that underpin VAWG as an outcome. Only two of the reviews included evaluations from LMICs, and only two of the reviews were rated highly using AMSTAR-2 (one was a review of bystander interventions—only in HICs—and one was a meta-analysis of college dating violence interventions that included LMICs), with the rest assessed as a mix of critically low to moderate quality.

A majority of the reviews in this category (12 of 16) were entirely focused on bystander interventions, published between 2013 and 2021, and largely evaluated in HICs (highlighting the lack of research on this issue in LMICs). Some of the bystander reviews included evaluations that showed changes both in VAWG^30 31^ and norms related to VAWG,^32^,^34^ highlighting how effective bystander interventions have been in HICs. However, more research is needed to confirm whether bystander interventions are indeed promising in reducing violence in HICs and to fill the evidence gap regarding interventions in LMICs. This review demonstrated an increased focus on bystander interventions in particular, strengthening the evidence base of effectiveness in HICs.

The RESPECT women framework has indicated that more evidence was needed in all settings to demonstrate whether infrastructure and transport interventions affect rates of VAWG. Similarly, more evidence was needed in LMICs to demonstrate effectiveness of whole-school interventions, and no evidence existed on whole-school interventions in HICs. This review did not generate sufficient evidence to change these evidence assessments.

### Child and adolescent abuse prevented

36 reviews addressed the prevention of child and adolescent abuse. Nearly all of these reviews had reductions in violence as an outcome (in this instance, violence defined as child sexual abuse, IPV and NPSV), and most had changes in norms that underpin VAWG as an outcome. 16 reviews showed a reduction in dating violence among adolescents. About half (21 of 36) of these reviews included evaluation studies from LMICs. These 36 reviews ranged in quality from critically low to high using the AMSTAR-2 rating system.

The child and adolescent abuse prevention strategy includes four categories of programmes: (1) home visitation and health worker outreach, (2) parenting interventions, (3) psychological support interventions for children who experience violence and who witness IPV and (4) life skills/school-based curriculum, rape and dating violence prevention training.

The primary shift in evidence within this strategy has been within life skills/school-based curriculum interventions, including rape and dating violence prevention training approaches. The RESPECT women framework indicated that there was conflicting evidence around these approaches in HICs and that more evidence was needed to demonstrate these approaches’ effectiveness in LMICs. This review strengthened the evidence in both settings, such that the evidence for life skills/school-based curriculum including rape and dating violence prevention approaches is no longer conflicting in HICs.^35^,^37^ It is categorised as ‘more evidence needed’ in HICs given that included reviews demonstrated improvements in intermediate outcomes related to violence. In LMICs, life-skills/school-based curriculum approaches have been recategorised to ‘promising’ in their ability to reduce VAWG in LMICs.^38 39^ A large portion of reviews in this category (20 of 36) were assessed to be of high or moderate quality, and most of the reviews from LMICs (11 of 17) were also of high or moderate quality. In other categories of interventions such as home visitation, parenting interventions, there is no meaningful shift in the evidence base on other approaches within this strategy (which, while promising in HICs, still needs more evidence in LMICs). These are important areas for future evaluations in LMICs.

### Transformed attitudes, beliefs and norms

Twenty-two reviews focus on interventions to transform attitudes, beliefs and norms that support violence. Therefore, all of them included changes in norms that support violence as an outcome, and all but one review included reductions in VAWG. In general, the reviews paint a positive picture of these interventions’ ability to reduce VAWG and change norms. All but 2 of these 22 reviews included evaluations in LMICs, and the reviews were of mixed quality, ranging from critically low to high on the AMSTAR-2 rating system.

Several programmatic approaches are included within this strategy, including (1) community mobilisation, (2) group-based workshops with women and men to promote changes in attitudes and norms, (3) social marketing or edutainment and group education, (4) group education with men and boys to change attitudes and norms and (5) stand-alone awareness campaigns/single component communications campaigns. While community mobilisation and group-based workshops with women and men were categorised as ‘promising’ in reducing VAWG in LMICs, other programmatic approaches (ie, social marketing or edutainment and group education, group education with men and boys, stand-alone awareness campaigns) were categorised as ‘more evidence needed’, having ‘conflicting evidence’ or ‘ineffective’ based on the systematic reviews available in 2019. Most of the evaluations^40^,^42^ of these approaches included in the present reviews demonstrated effectiveness only in changing norms and attitudes, not VAWG.

In the present research, there are now more reviews with evaluations of interventions of group education with men and boys to change attitudes and norms in LMICs. Evidence from this review indicates that these approaches in LMICs have now demonstrated positive shifts in norms through several new evaluations. More evidence is needed to determine whether these approaches are effective in reducing VAWG. More research is needed on community mobilisation programmes in HICs, as these approaches are demonstrating success in reducing VAWG in LMIC settings.

## Discussion

New evidence has emerged from the present review of reviews that can inform the evidence underpinning the RESPECT women framework strategies since its publication in 2019. Many more reviews were captured here than were included in the previous review of reviews^3^ (one of the main sources informing the RESPECT women framework), reflecting an increase in research attention to the issue of VAWG prevention.

This review particularly highlights the proliferation of evidence focused on perpetrator interventions under the services strategy and bystander interventions as part of environments made safe strategy, with both approaches often evaluated only in HICs. In LMICs, by contrast, there has been an increase in the number of reviews focusing on the transformation of gender attitudes, beliefs and norms strategy and the child and adolescent abuse prevention strategy. The bulk of the evidence generated in LMICs is coming from sub-Saharan Africa and South Asia.

This review strengthens the overall body and quality of evidence for some interventions to empower women and girls (specifically those combined with microfinance and gender empowerment training), which are working to reduce VAWG. The present findings warrant additional investment in these approaches given that the quality of evidence is moderate to high. Under the services category, empowerment counselling interventions and psychological that support access to services (ie, advocacy) also now demonstrate promising effects on VAWG reduction in LMICs. Most of the reviews in this category were rated to be of moderate or high quality, indicating additional investment in these approaches is also warranted.

The evidence base for life skills/school-based curricula, including rape and dating violence prevention training, has improved under the category of child and adolescent abuse prevention with most reviews in this category of moderate to high quality. Another category of interventions with more evidence of effectiveness is group education with men and boys to change attitudes and norms in LMICs.

Finally, perpetrator interventions consistently fail to demonstrate effectiveness (though they remain a key focus of investment and evaluation, especially in HICs). Investments in prevention should shift away from perpetrator interventions and towards other approaches that are demonstrating reductions in VAWG.

The present systematic review of reviews has several limitations, owing mostly to the substantial diversity of the included articles. First, the quality of reviews varied widely, making it difficult to draw summary conclusions about the state of the VAWG prevention evidence based on the AMSTAR-2 ratings. This may be due in part to the nature of the AMSTAR-2 tool, which is a one-size-fits-all instrument for a set of articles that varied widely in scope and methods. The imperfect nature of AMSTAR-2 as a systematic review of reviews quality assessment tool is a limitation. Second, we included reviews that contained experimental and/or quasi-experimental evaluations. Findings from non-experimental evidence (eg, evidence of impact of policy-level changes) have been omitted, limiting our ability to reflect on large-scale, societal-level impacts that may result from policy changes. Evidence has demonstrated that these kinds of policy-level changes can have a significant impact on rates of VAWG (eg, in Nicaragua, where policy changes contributed to a greater than 60% reduction in women’s lifetime experiences of violence over a 20-year period from 1995 to 2015).^43^ Third, there was substantial heterogeneity among the data summarised in included reviews, limiting our ability to conduct any kind of meta-analysis or draw summary conclusions with included data. The fact that no additional articles were yielded from searches conducted in languages other than English points to the possibility that these translated terms were inadequate, or there may be articles in other languages we missed. Finally, given that the search was conducted in April 2022, the present article reflects an evidence synthesis that, at the time of publication, is somewhat aged.

This review reveals several remaining gaps in the current VAWG evidence base. More research is needed on the impact of certain programmatic approaches on subpopulations at a higher risk of violence—be it adolescent girls, indigenous women, women with disabilities or other particularly marginalised groups. Future research should consider for whom, and under what circumstances, interventions are most effective in decreasing VAWG. For example, preliminary analysis of evaluations of poverty reduction strategies highlighted positive results in some subgroups, not necessarily in all women. Though a preponderance of the reviews captured in the present review continues to be under the services ensured strategy (which has dominated the field of VAWG prevention), more investments are still needed in evaluating the range of service interventions beyond perpetrator interventions. Traditional public health interventions have often followed a trajectory of being evaluated in HICs and then adapted for LMICs. In the VAWG prevention field, several programmatic approaches are demonstrating effectiveness in LMICs that could be adapted and evaluated in HICs (eg, community mobilisation programming, inheritance and asset ownership policies and one-stop crisis centres), allowing for reverse learning of innovations from low resource settings to high resource ones. At the same time, several successfully evaluated strategies that are receiving extensive research attention in HICs have yet to be evaluated in LMICs. A key example is bystander interventions, which have been extensively evaluated and are demonstrating some effectiveness in HICs and should be adapted to and tested in LMICs.

Understanding what works to prevent VAWG, and in which settings and populations, has important implications for policy-makers, donors and programme implementers. Those implementing these interventions are often local women’s rights/feminist organisations at the frontlines of innovations in the prevention field. Building on the learnings from the last 5–10 years, the next round of investments in VAWG prevention field should be geared towards scaling up promising prevention strategies. This might include exploring how interventions can be better integrated into broader public health and social welfare systems. This review of reviews will not only contribute to updating the evidence base for the RESPECT women framework but also seeks to guide new and future initiatives that aim to scale up prevention of VAWG with the latest evidence on what works.

## Supplementary material

10.1136/bmjph-2024-001126 online supplemental file 1

10.1136/bmjph-2024-001126 online supplemental file 2

10.1136/bmjph-2024-001126 online supplemental file 3

10.1136/bmjph-2024-001126 online supplemental file 4

## Data Availability

Data sharing not applicable as no datasets generated and/or analysed for this study.
