# Supplementary material for: Interventions to prevent violence against women and girls globally: a global systematic review of reviews to update the RESPECT women framework
Source: BMJ Public Health. 2025 Jan 20;3(1):e001126. doi: 10.1136/bmjph-2024-001126 (PMC11816861; doi:10.1136/bmjph-2024-001126)
Supplement: online supplemental file 1 [file bmjph-3-1-s001.pdf]

Appendix 1: Detailed information on RESPECT women framework strategies

Relationship skills strengthened

Interventions in this RESPECT Framework category aim to improve the communication, conflict management, and decision-making skills amongst men, women, and couples to promote equitable power in relationships.[17.] Since women are more likely to experience violence perpetrated by their current or former intimate partners, implementing interventions that can protect women from the unequal gender and power differentials in relationships is crucial.[18] Women that are in relationships where the couples have strong communication, and gender equitable values, are less likely to be victims of IPV.[25] Interventions in this category include activities like educational workshops focused on fixing power imbalances, or couples counselling.

Empowerment of women

Empowerment interventions aim to empower women socially and economically through microfinance and empowerment training interventions, and mentorship to build skills in self-efficacy and decision-making[17]. Educational trainings and mentoring, interventions to improve female inheritance and asset ownership policies, and economic interventions with holistic personal empowerment components are all interventions that fit within the Empowerment of women category.

Services ensured

Of all the women that experience violence, only 40% of them seek out any form of outside help.[26] Safe, confidential, and high quality services for survivors of violence are essential in preventing violence and mitigating the consequences of violence.[20] Improved reporting and social services can create entry points through which violence can be prevented, and improved police, legal and health services can play a direct role in reducing the negative effects of perpetrated violence. All these services, when they center the rights of women and dignity of survivors, can provide a continuum of care that minimizes harm to women and girls. The services available to survivors of violence (including police, legal, health, and social support) are included in this category of interventions.

Poverty reduced

Women living in poverty are at increased risk of IPV and non-partner sexual violence (NPSV). Women and girls living in poverty are more likely to have weak legal and support services available to them, and are more likely to have additional demographic risk factors for violence such as lower education and socioeconomic class. Violence experienced by women can also lead to financial costs (health, legal, and indirect social costs) further entrenching women in poverty. These risk factors are further exacerbated in low- and middle-income countries (LMICs) where the overall governmental and structural support systems may be weakened.[27] Economic interventions to reduce poverty in LMICs have shown some past success. These interventions include direct cash transfers to women, which can be unconditional or conditional (dependent on

participation in other aspects of a program like child nutrition or home visits).[21] Other examples of interventions to reduce poverty are labor force interventions, which improve employment opportunities for women or provide skills based training. Microfinance or savings interventions that focus only women's economic growth through building assets (without additional components) also fall under this category of poverty-reducing interventions.

Child and adolescent abuse prevented

Violence against women and violence against children have common risk factors and often co-occur, as violence is often occurring in the same households, and is a result of the same patriarchal values that normalize violence.[28] This violence is not only perpetrated by male family members, as women who are survivors of violence themselves are more likely to use violence to discipline their children than women who are not.[29] Violence experienced in childhood is a predictor of future violence perpetration for both girls and boys. The goal interventions in this category is to nurture family relationships, reduce corporal punishment (both in schools and by care-givers), and reduce childhood sexual abuse. Interventions to prevent violence against children include both preventative and remedial methods. These may include parenting interventions to improve the relationship between the child and caregiver, and/or to promote gender equality and safer parenting methods. Home visitation programs for women who are at risk of IPV to identify and mitigate child abuse are also included in this category. Interventions may include school- or community-based curricula for young people to identify and prevent dating violence and promoting gender equality.[23] Psychological support interventions for children to address trauma are included in this group. Additional programs to prevent violence against children are further detailed by the INSPIRE framework.[30]

Transformed attitudes, beliefs, and norms

Gender discriminatory attitudes and beliefs are key drivers of violence against women and girls.[13] Interventions in this category promote positive attitudes and norms around gender that can achieve sustainable reductions in violence and create safer communities[24]. Interventions that are a part of this category aim to challenge these attitudes and beliefs that drive violence against women and girls by changing the status-quo. These interventions work with individuals, families, and entire communities. Interventions within this group include community mobilization approaches that engage activists who live and work in the community. These interventions can include informal community activities led by community activists, and social marketing campaigns or "edutainment" programs that integrate lessons around gender equitable attitudes within popular media[24]. Other kinds of interventions in this category include peer-based workshops and educational programs that encourage discussions around gender norms, and group education with men and boys to discuss masculinity and gender equality.
