# Supplementary material for: Interventions to prevent violence against women and girls globally: a global systematic review of reviews to update the RESPECT women framework
Source: BMJ Public Health. 2025 Jan 20;3(1):e001126. doi: 10.1136/bmjph-2024-001126 (PMC11816861; doi:10.1136/bmjph-2024-001126)
Supplement: online supplemental file 2 [file bmjph-3-1-s002.pdf]

Appendix 2: Search terms

Violence outcome	violence against girls OR violence against women OR VAW* OR domestic violence OR GBV OR gender violence OR gender-based violence OR femicide OR feminicide OR human trafficking OR trafficking of persons OR partner violence OR abuse of women OR wife abuse OR abuse of wives OR wife battering OR battering of wives OR battering of women OR spouse abuse OR family violence OR murdering of women OR homicides of women OR honor killing OR acid attack OR acid throwing OR missing women OR missing girls OR widow burning OR stoning of women OR rape OR sexual violence OR sexual abuse OR sexual assault OR sexual harassment OR coerced sex OR unwanted sex OR unwanted fondling OR unwanted touching OR sex trafficking OR sexual exploitation OR forced prostitution OR sexual slavery
Article type	review OR meta-analysis OR overview OR summary OR synthesis
Intervention type	prevent* OR intervention* OR program* OR approaches OR trial* OR evaluation* OR response* OR evidence OR impact* OR effect* OR efficacy OR what works
