# Supplementary material for: Interventions to prevent violence against women and girls globally: a global systematic review of reviews to update the RESPECT women framework
Source: BMJ Public Health. 2025 Jan 20;3(1):e001126. doi: 10.1136/bmjph-2024-001126 (PMC11816861; doi:10.1136/bmjph-2024-001126)
Supplement: online supplemental file 3 [file bmjph-3-1-s003.pdf]

Author year	Aims and Population	Includes LMICS	Type of Review	# of studies	Results	Quality Score
Akoensi, et al 2013	This review examined the effectiveness of interventions designed to alter the attitudes and/or behaviors of domestically violent perpetrators - male and female. Domestic violence perpetrators included those convicted of a domestic violence offence or those who had initiated treatment to address self-reported violent behavior. The review was	no	Systematic Review	12	No definitive conclusions drawn regarding effectiveness of interventions. Unclear on what works best, believe this to be the case globally in regards to domestic violence research.	High
Ali, et al 2021	This review's objective was to evaluate the accuracy and effectiveness of IPV screening tools and subsequent interventions in non-risk settings. Reviews that included addressed IPV affecting men and women aged 16 and older were included.	no	Non-Systematic Review	23	Simple screening tools and subsequent interventions found effective. HARK, PSQ, and HURT proved to be particularly successful screening tools as well as multiple types of interventions. Yet, evidence was insufficient to suggest adapting UK guidelines.	NA
Alvarez, et al 2016	This review sought to evaluate elements of interventions that addressed IPV in Latina women. Included studies samples had to be at least 25% Latino (especially Latina women) and aged 18-25. All studies evaluated were conducted in the United States.	no	Systematic Review	4	Found that interventions targeting IPV within the Latino population were sparse. Drawing comparisons made challenging by heterogeneity. Studies that reported reduced IPV had some common components: theoretical framework, multiple sessions, group settings, focus on improving relationships with partners and family.	High
Anderson, et al 2013	This review examined interventions that focused on both IPV and HIV in health care and community based contexts. Interventions assessed were conducted in South Africa, Tanzania, and Nigeria. The goal of the review was to assess which interventions could potentially be implemented by nurses in Sub-Saharan Africa.	yes	Systematic Review	17	Bigger dynamic community based interventions associated with positive impacts on social norms related to women's empowerment. Educational interventions appear to have short-term improvement in HIV knowledge and behavioral practices. More research needed to examine HIV/IPV interventions in healthcare settings.	Low
Anderson, et al 2021	The goal of this review was to evaluate web-based and mobile health interventions that focused on IPV victimization prevention. The vast majority of studies were conducted in the US with only one based in an LMIC. Most studies analyzed included only women but some did include men in heterosexual relationships.	yes	Systematic Review	31	Evidence of efficacy in IPV victimization prevention of mHealth interventions compared to non-web based interventions is limited - more research needed. mHealth found to be uniquely effective in healthcare settings, mobile phone platforms, and facilitating victim's connection with health care providers. Ability to customize interventions to victim needs without significant	High
Arango, et al 2014	This systematic review of reviews broadly synthesized global evidence on what works to prevent violence against women and girls. VAWG was categorized as IPV, non-partner sexual abuse, harmful traditional practices, human trafficking, and child sexual abuse. The majority of reviews discussed were conducted in high income countries.	yes	Systematic Review of Reviews	58	Some evaluations of psychosocial support showed decrease in violence in high-income settings. In LMICs, several primary prevention measures resulted in a reduction of VAWG. Most successful interventions considered risk factors for violence, administered dynamic and cohesive approaches, and engaged with several stakeholders.	NA
Araujo, et al 2021	This review focused on maladaptive beliefs in adolescent relationships, specifically if/how these beliefs constrain these relationships, the types of beliefs, the impact these beliefs can have on violent offenses, and whether interventions can alter these beliefs. Studies analyzed centered on young adults and were all based in high income countries.	no	Systematic Review	15	Results indicate maladaptive behaviors can legitimize anti-social tendencies and violence (sexual, physical, GBV, IPV, or domestic.) Prompt identification of beliefs and understanding their impact on maladaptive behaviors enable more effective interventions to reduce violence and better relationship quality. Gender differences in presence and manifestation of beliefs and behaviors,	Critically low
Arce, et al 2020	This meta-analysis evaluated the effectiveness of batterer intervention programs. The outcome of interest was recidivism. There was no specific geographic or population focus.	yes	Systematic Review	25	Meta-analysis results showed positive, significant and of medium magnitude effect size for batterer interventions but this was not generalizable. Efficacy measuring in couples reports was null but those in official records proved significant and positive. Higher effects observed with cognitive behavioral treatment programs and longer interventions.	Moderate
Armenti, et al 2016	The goal of this review was to evaluate current US based court-mandated male-only IPV Duluth and CBT interventions and compare these to conjoint (couples based) interventions for court-mandated offenders. The main idea was that conjoint interventions can address the shortcomings of male-only interventions and benefit some partner-violent couples.	yes	Systematic Review	8	Evidence suggested that conjoint communication and relationship skills programs can be an alternative for screened subset of couples that experience predominantly situational violence. Conjoint interventions not advisable for various types and manifestations of violence.	Low
Arroyo, et al 2017	This review evaluated the effect size of short-term psychotherapy interventions provided to adult IPV survivors. Targeted outcomes included PTSD, self-esteem, depression, general distress, life functioning, substance use, emotional wellbeing, safety, and interpersonal violence.	yes	Meta-Analysis	21	Most short-term psychotherapy interventions had large effect sizes and improved outcomes for IPV survivors. CBT interventions that specifically focused on IPV victims were found to be the most effective.	Critically low
Awolaran, et al 2022	This review's goal was to synthesize interventions to reduce IPV in LMICs. Interventions targeted at either or both females and males but key outcome had to be prevention of IPV against women. Interventions had to target IPV attitudes/behavior either by primary or secondary prevention.	yes	Meta-Analysis	48	Results on the effectiveness of interventions on participants' attitudes/behavior regarding IPV were small and non-significant. Heterogeneity in intervention approaches made comparisons difficult.	High
Babaee, et al 2021	This review included evaluations of population-based domestic violence interventions. Outcomes of interest included occurrence and repetition of multiple types of violence. Intervention types also varied.	yes	Systematic Review	18	Unable to draw conclusions on intervention effectiveness due to poor quality rating of included studies.	High
Bacchus, et al 2017	This review identified interventions that measured outcomes for IPV and child maltreatment and evaluated their effectiveness. Interventions had to be conducted in LMICs. Outcomes included changes in knowledge/attitudes towards IPV and child maltreatment and/or reported outcomes of both.	yes	Non-Systematic Review	6	Unable to evaluate effectiveness accurately due to insufficient evidence and methodological weaknesses. Suggested that increased coherence between IPV and child maltreatment programs is viable, especially in community-based interventions that target parents.	NA
Bair-Merritt, et al 2014	This review summarized and evaluated primary care-focused IPV interventions. No geographic criteria but most interventions were conducted in the United States. Outcomes included IPV reduction, physical and emotional health, safety-promoting behaviors, use of IPV and community-based referrals/resources.	yes	Systematic Review	17	Majority of studies indicated improved outcomes subsequent to primary care IPV interventions. IPV/community referrals were the most common positively affected outcome. Successful interventions facilitated self-efficacy and empowerment, access to IPV resource, brief non-physician delivery models.	Critically low
Baptista, et al 2021	This narrative review examined the effectiveness of interventions targeting heterosexual male perpetrators of violence against women. Types of interventions examined include cognitive behavioral therapy, thematic discussions, group discussions, and motivational strategies. Studies had no geographic focus.	yes	Non-Systematic Review	9	Poor motivation was associated with higher chances of leaving the program and relapsing - most who gave up cited feelings of injustice. Participants regarded facilitators and discussions as very important. None of the interventions were associated with lasting change.	NA
Baranov, et al 2021	This analysis evaluated the effects of cash transfer programs on IPV in LMICs. Authors also explored various theories within a household bargaining context.	yes	Meta-Analysis	15	Majority of studies reported that cash transfers either positively affect IPV or don't affect it at all. However, some studies showed an increase in IPV occurrence post-intervention. Significant evidence found on physical and emotional violence and controlling behaviors.	Low

Author year	Aims and Population	Includes LMICS	Type of Review	# of studies	Results	Quality Score
Barnett, et al 2018	The main focus of this review of reviews was recidivism and proven reoffending outcomes. This reviews included evaluations of interventions geared towards reducing violence, domestic violence, sexual offending, drug misuse, driving under the influence, and general reoffending. The vast majority of reviews discussed were conducted in high income	no	Systematic Review of Reviews	21	Interventions that are punitive or deterrent in nature less likely to reduce reoffending, and can make people more likely to commit crimes if administered without rehabilitative support. Approaches that are discipline-based are not effective at reducing reoffending if they don't encompass rehabilitative support. Added supervision of those being surveilled in the community	NA
Beek, et al 2018	This study reviewed research on the effect of measured recidivism (repeated arrest/conviction) of treatments targeting adolescents who committed sexual offenses. Mean age of intervention sample had to be between 12-18.	no	Meta-Analysis	14	Moderate effect size that suggested reduction in recidivism no longer significant after controlling for publication bias. Treatment groups found equally effective for all types of recidivism. Interventions with shorter follow-up times found had larger effect sizes.	High
Bourey, et al 2015	This study reviewed the impact of structural interventions to IPV in LMICs. Structural interventions defined as those which target economic, physical, politico-legal, or social systems that may pose an IPV risk. No population restrictions specified.	yes	Systematic review	20	Economic interventions reviewed found less likelihood of controlling behaviors, positive impact on economic well being, and improved relationship quality. Social interventions were associated with reduced physical, psychological, sexual, and physical or psychological IPV, increased support of equitable gender norms, amongst other positive outcomes. Combined economic and social	High
Bowring, et al 2017	This review of reviews examined reviews that focused on young people and reported on sexual and reproductive health outcomes (pregnancy, sexually transmissible infections, condoms/contraceptive use, risky sexual behavior, sexual healthcare access or IPV.) Reviews synthesized primarily based in high income countries.	no	Systematic Review of Reviews	66	Successful interventions had these characteristics in common: spanned a longer period of time/included multiple sessions, executed in multiple settings/levels, included parental involvement, customized to relevant populations, included skills building, and blended more than one component (i.e. education and skills building.) Whether interventions that focus on more	NA
Brooks, et al 2014	This review, comissioned by the Scottish Government, focused on interventions with perpetrators to reduce reoffending. Interventions reviewed include justice responses to domestic abuse, rape, and sexual assault; domestic abuse perpetrator programmes; government responses to stalking, and commercial sexual exploitation. The review has no specific population or geographic focus.	no	Non-Systematic Review	11	The most effective perpetrator interventions engaged men, incorporated risk management, and effectively coordinated between services and the course. Early and repeated police intervention with perpetrators seems to be more effective than arrest. Multi-disciplinary, community-oriented approaches were most effective in addressing stalking. Domestic violence protection orders demonstrated mixed effects. More evidence is needed on the effectiveness of special domestic	NA
Buller, et al 2018	This review's goal was to examine how cash transfers impact IPV in low and middle income countries. Included studies evaluated the effect of cash transfers on emotional, physical, and/or sexual IPV. The vast majority of programs evaluated targeted women as the main recipients of the cash transfer intervention.	yes	Non-Systematic Review	14	Results indicate that cash transfers can reduce IPV and evidence not associated with increases in IPV. Cash transfers can impact types of violence differently, stronger results for reduction of physical/sexual violence. Found evidence to corroborate notion that cash transfers can positively impact economic security, emotional well-being, conflict in the home, and women's	NA
Carr, et al 2019	The goal of this review was to assess the effectiveness of couple therapy, family therapy, and systemic interventions for adults with a range of relationship and/or mental health problems. Systemic practice defined as couple and family therapy and other family-based interventions. Adult mental health and relationship problems included IPV, psychological	no	Non-Systematic Review	2	Evidence suggested systemic interventions were effective in reducing a variety of mental health and relationship issues. This applies to systemic interventions either alone or in conjunction with another program seeking to address similar issues.	NA
Chen, et al 2022	This analysis evaluated the effects of digital health interventions on preventing and reducing unintentional injury, violence, and suicide. Studies were not limited by participant age, geographic focus or intervention content.	no	Meta-Analysis	34	Based on the included studies, analysis found a small but significant positive effect size at post-intervention which is comparable to traditional interventions of the same vein. Digital interventions directly addressing violence and suicide tended to be more effective. Digital interventions found to be more effective at knowledge and skill building rather than reduction of	High
Cheng, et al 2021	This review and analysis focused on the effectiveness of batterer intervention programs in decreasing recidivism in domestic violence. Studies had to be conducted in North America. The outcomes of interest were domestic violence and general offense recidivism reported by the criminal justice, system and IPV perpetration reported by the survivor.	no	Meta-Analysis	17	Results indicated that batterer intervention programs were effective at reducing domestic violence and general offense recidivism when reported by the criminal justice system but not when reported by the survivor. Effectiveness varied depending on study design - more rigorous interventions were associated with less positive impact. According to survivor reports, no	Critically low
Choo, et al 2015	Studies reviewed measured the effectiveness of emergency department IPV interventions. All interventions were conducted in North America.	no	Systematic Review	3	According to the two evaluated studies, IPV related outcomes improved for both treatment and control groups. Insufficient evidence precluded a robust analysis of emergency department IPV interventions.	Critically low
Cleaver, et al 2019	The aim was to identify and evaluate multi-agency early interventions targeting IPV in the United Kingdom. Interventions included primarily focused on heterosexual female domestic abuse survivor and male perpetrators of abuse. At least two independent agencies had to be involved in the intervention.	no	Systematic Review	22	Interventions that adopt an advocacy approach and/or work with voluntary or statutory services more successful in encouraging victims to report abuse. Early intervention considered an effective preventative strategy - especially among children and young people. Early domestic abuse intervention reliant on survivors having access to appropriate services.	Critically low
Cordier, et al 2021	The purpose was to evaluate the effectiveness of protection orders in reducing domestic violence recidivism, compare rates reported by victims and police, and identify factors that may influence recidivism. Studies included female victims, male offenders, and female offenders. All studies but one conducted in the United States.	no	Meta-Analysis	25	Violation/recidivism rates higher for victim reports. Violation of protection order rates lower when orders were used alongside arrests. Violation rates lower for offenders with no histories of previous violence related arrests, did not engage in stalking, and couples with more income.	High
Cork, et al 2020	This review identified interventions designed to prevent or reduce IPV in Sub-Saharan Africa. Included studies had to be published RCTs. Intervention types were varied.	yes	Systematic Review	15	Studies indicated that interventions can reduce IPV-related behaviors and attitudes. Interventions more successful at reducing sexual IPV rather than physical IPV. Successful interventions tended to have longer follow-up periods, had IPV as their primary aim, were community or multi-level based.	High
Crane, et al 2017	This review evaluated integrated IPV interventions which focus on comorbid risk factors in addition to IPV behaviors. Psychological comorbid risk factors include substance abuse and trauma. Multiple intervention designs reviewed.	no	Non-Systematic Review	13	Integrated treatment models appeared to significantly improve treatment compliance and reduce acts of IPV. A motivational interviewing component proved very important. Greater variability in integrated treatment options needed to address heterogeneity of perpreator population.	NA
Crooks, et al 2019	This review included studies of GBV prevention interventions targeting girls and young women. Primary and selective prevention approaches were included. Majority of research discussed in North American context.	yes	Non-Systematic Review	104	Identified knowledge gaps regarding GBV prevention approaches that don't only work for cisgender, heterosexual, white young people. Need more flexible and participatory research designs. Reconsider what constitutes as evidence.	NA
Cunha, et al 2014	Interventions analyzed centered on batterer treatment programs for heterosexual male offenders. Majority of interventions evaluated were conducted in the North America and Europe but a couple of LMICs included. Program outcomes included skills promotion, anger management, equality promotion, amongst others of a similar nature.	no	Systematic Review	36	Findings confirm that effectiveness of batterer intervention programs remains controversial. Results regarding efficacy of different programs remains inconsistent. Argument made that small reductions in recidivism can have significant social impacts.	Low

Author year	Aims and Population	Includes LMICS	Type of Review	# of studies	Results	Quality Score
Daley, et al 2020	This review investigated evidence of interventions for women who report domestic violence during and after pregnancy in LMICs. Outcomes of interest were reduction in frequency/severity of domestic violence, improved physical, psychological, and/or social health. Women during pregnancy, childbirth, or up to 12 months postnatal were the target	yes	Systematic Review	6	Evidence found was low-moderate quality. Some improvement found in women's quality of life, use of safety behaviors, family and social support, access to community resources, increased use of referral services, and reduced maternal depression. Screening, referral, and supportive counselling likely to help.	High
de Koker, et al 2014	This review evaluated trials of interventions to reduce physical, sexual, and psychological violence among young people. Participants in included studies were between 10 and 19 years old. All but one trial were conducted in North America.	yes	Systematic Review	9	Positive intervention effects found on IPV perpetration and victimization. Effective interventions tended to be longer, based in school and community settings, and focused on key people in the adolescent's environment. All trials had quality issues.	High
De la Rue, et al 2014	The main goal of this review was to synthesize and evaluate programs that sought to reduce or prevent teen dating or sexual violence. Outcomes of interest included attitudes and beliefs, dating violence perpetration, or dating violence victimization. All programs were conducted in the United States with teens in grades 4-12 (approx. 9-18.)	no	Meta-Analysis	23	Students in the intervention condition showed improved violence related knowledge, increased use of conflict resolution skills, endorsed less violent attitudes, and were less accepting of rape myths. Results suggested that programs are not impacting violence perpetration and victimization to a significant extent.	High
De la Rue, et al 2017	This meta-analysis focused on more recent school-based interventions designed to prevent or reduce dating violence in adolescent relationships. Included studies were conducted in the United States and children were between 11-18. Primary outcomes were knowledge, attitudes, and behaviors.	no	Meta-Analysis	23	Results indicated that school-based violence prevention programs had a significant impact on adolescent dating violence knowledge and attitudes. At follow-up, students showed moderate increase in knowledge, lower rape myth acceptance, and improved conflict resolution skills. Reductions in the perpetration of dating violence victimization were minimal and not sustained.	Moderate
de Oliveira, et al 2016	This review evaluated the methodology of interventions addressing IPV among adolescents. Global focus but most studies were conducted in North America. Studies were conducted in various settings and had varying methodologies.	yes	Non-Systematic Review	30	The authors concluded that the studies did not address violence in the context of gender and adolescence to a satisfactory level. Dimensions beyond changes in behavior and attitudes such as social structures were not considered.	NA
DeGue, et al 2014	This review summarized and evaluated evidence on primary prevention strategies for sexual violence perpetration. Most strategies were brief psycho-educational interventions focused on changing attitudes and behaviors. Study population included mostly school-aged children and college students.	no	Systematic Review	140	Only three strategies found effective: "Safe Dates," "Shifting Boundaries," and funding related to the 1994 United States "Violence Against Women Act." Other interventions had null or harmful effects.	Critically low
Denhard, et al 2020	This review addressed whether and to what extent alcohol use interventions in college campuses affect sexual assault outcomes. All interventions were conducted in the United States. Interventions had to measure outcomes related to sexual assault victimization or perpetration or bystander behaviors.	no	Non-Systematic Review	7	Researchers only found 7 studies that explored the relationship between alcohol use and sexual assault. Most studies indicated a reduction in sexual assault outcomes. Alcohol use interventions can be an effective strategy for reducing sexual assault outcomes.	NA
Dowling, et al 2018	This study reviewed research on the use and impact of protection orders related to domestic violence. All studies were conducted in Australia, New Zealand, the United States, the United Kingdom, or Canada. The primary outcome was domestic violence re-victimization.	no	Meta-Analysis	63	According to results, victims who had a protection order were significantly less likely to experience re-victimization. Despite its significance the effect size of this result was small. Protection orders found to be more effective when the victim was able to be more independent and had less ties to the perpetrator.	Low
Dowling, et al 2018a	The focus of this review was police-led responses to IPV. It examined six fields of police response: workforce development, police reporting, first response, prevention of further domestic violence, investigative responses and charging of perpetrators. Eligible studies were based in Australia, New Zealand, the United States, the United Kingdom, or Canada.	no	Systematic Review	346	Police efforts can have a positive impact on the odds of further violence, victim satisfaction and wellbeing, and criminal justice system outcomes. Measures to improve police responses further should focus on deploying responses at the incidents, victims and perpetrators where they are likely to have the largest impact; developing the workforce; using new technologies and	Low
Dworkin, et al 2013	This review analyzed gender-transformative HIV and violence interventions that targeted heterosexually-active men and boys. Interventions were not limited to a geographic scope. Outcomes of interest included of STI and HIV infections, VAW, sexual risk behavior, and inequitable gender norms.	yes	Systematic Review	15	Review suggested that gender-transformative HIV and violence interventions can significantly increase protective sexual behaviors, change harmful gender attitudes, prevent sexual and physical violence against women, and reduce HIV/STI infections.	Low
Eckhardt, et al 2013	This review evaluated the effectiveness of IPV interventions for perpetrators and survivors. A variety of intervention types and settings were included. Primary outcome was violence recidivism.	no	Narrative Review	61	Interventions for perpetrators show inconsistent results regarding their ability to reduce rates of IPV. Interventions for IPV survivors, structured counseling programs appeared to reduce victimization. Difficult to ascertain whether interventions for survivors contribute to longer term IPV reduction.	NA
Eggers, et al 2020	This systematic review and meta-analysis investigated the impact of female economic empowerment interventions on IPV. Most included studies were carried out in Sub-Saharan Africa. Principal outcomes were sexual, physical, emotional violence and controlling behaviors.	yes	Meta-Analysis	20	Evidence suggested that in most settings economic empowerment interventions were associated with a reduction in IPV. The reduction effect size was small but significant. Larger effects were associated with more generous cash-transfer and microfinance interventions.	High
El Morr, et al 2020	This review evaluated research on ICT-based IPV interventions. Intervention recipients were women who experience domestic violence or any form of IPV. Most studies reported on were conducted in the United States.	no	Systematic Review	25	Evidence suggested that ICT interventions were most effective at screening, disclosure, and prevention. Yet, evidence base is insufficient. Need for more women-centered ICT design in IPV programs.	Moderate
Ellsberg, et al 2015	This review examined interventions aimed at reducing violence against women and girls. The majority of studies were based in high income countries. Most interventions evaluated focused on responses to violence.	yes	Systematic Review	22	Found that home-visitation, advocacy, and women centered interventions can reduce the likelihood of further victimization but there was less significant evidence associated with preventative interventions geared towards perpetrators. In LMICs, strong initial evidence found of the positive impact of women and men group training, community mobilization interventions,	Low
Emezue, et al 2021	This reviewed focused on batterer intervention programs that targeted immigrant male batterers and sought to identify effective components among these programs. Most studies evaluated were conducted in the United States. Program approaches varied.	no	Systematic review	8	Addressing and discussing cultural expressions of masculinity produced short-term improvements in changing IPV related attitudes. Findings with immigrant batterers not disaggregated by sub-group so results are broad generalizations. Methodological challenges affected program designs and implementations significantly.	Moderate
Esquivel-Santoveña, et al 2016	This review focused on the components and effectiveness of domestic violence perpetrator programs in Latin America and the Caribbean. To be included, programs had to focus primarily on perpetrators. The primary outcome was rates of recidivism.	yes	Systematic Review	12	Recidivism rates gathered varied widely with some as high as 90%. Evidence indicated that domestic violence perpetrator programs in Latin America and the Caribbean are in their earliest stages.	Low

Author year	Aims and Population	Includes LMICS	Type of Review	# of studies	Results	Quality Score
Evans, et al 2019	This review's goal was to evaluate the effectiveness of school-based bystander sexual violence interventions in the United States. Included studies had to evaluate interventions that targeted first-year college students and aimed to reduce the incident of sexual violence on college campuses.	no	Systematic Review	11	The evidence suggested bystander interventions were effective in increasing bystander confidence but most of the other results were insignificant. Programs were not successful in having a lasting impact on bystander behavior, efficacy, and willingness to intervene.	Moderate
Fagan, et al 2013	This review evaluated studies that reported on interventions targeting the perpetration of physical or sexual violence by youth aged 0-18. Violence was interpreted as interpersonal aggressive behavior where harm is attempted and/or inflicted by one person upon another. Included studies were conducted in the US and Canada.	no	Systematic Review	17	Interventions that focus on reduction of risk factors and improvement of promotive and protective factors found to significantly reduce violent behavior. Interventions designed to address whole communities and selected high risk individuals are both effective, design should be context-specific. Continued evaluation needed to ascertain effects on diverse communities.	Critically low
Fellmeth, et al 2013	The objective of this review was to evaluate the effectiveness of educational and skills-based interventions designed to prevent dating violence/IPV in adolescents and young adults. The age range for inclusion was between 12-25. All except for one study were conducted in the United States.	no	Systematic Review	38	Results indicated that there was no significant evidence that the interventions decreased rates of relationship violence. There was also no convincing evidence that they improved participants' attitudes, behaviours and skills related to relationship violence. Participant's knowledge about relationship slightly improved.	High
Feltner, et al 2018	This review examined the evidence on IPV screening and interventions, elder abuse, and abuse of vulnerable adults. The review was carried out to inform the US Preventive Services Task Force. Main outcomes were abuse, neglect, or comorbidity caused by abuse.	no	Systematic Review	30	IPV screening interventions did not show a reduction in IPV or improvement in quality of life over 3-18 months. Limited evidence suggested home visiting and behavioral counselling interventions that address multiple risk factors can contribute to reduced IPV among pregnant and postpartum women. No studies evaluated screenings or programs for elder abuse or abuse of vulnerable	High
Fenton, et al 2016	This report summarized evidence on bystander programs designed to reduce violence against women in university settings. The majority of studies were conducted in the United States.	no	Non-Systematic Review	12	Statistically significant positive results found for participants in bystander interventions across cognitive and attitudinal measures. Evidence suggested that replication of the interventions conducted in the United States could help address rates of violence in British universities.	NA
Fernandez-Fernandez, et al 2022	The goal of this review and meta-analysis was to analyze the effectiveness of interventions targeting gender abusers. Included interventions had to have a sample of gender abusers who were at least 18. Main outcome of interest was recidivism.	no	Meta-Analysis	26	Analysis showed that interventions that lasted fewer than 16 weeks/sessions and follow-up periods greater than or equal to 12 months had a positive, significant, and nearly moderate effect size. A positive, significant, and moderate effect size was found for interventions using cognitive-behavioral therapy. Despite this, no definite conclusions could be drawn about the effectiveness	Critically low
Ferriera, et al 2019	This review identified evidence on the care/treatment of perpetrators of family violence. Population was not restricted by sex or age. Main categories of focus were intervening factors for intra-family conflict and successful measures of rehabilitation of perpetrators.	no	Non-Systematic Review	15	Evidence suggested that inter-generational experiences and occurrence of violence in childhood may be intervening factors for aggressive family behavior. Some measures were found successful in the care of perpetrators: mindfulness meditation, individual counseling, improvement in sleep, participation in prevention programs, and psychotherapy.	NA
Feyissa, et al 2015	The objective of this review was to assess the effectiveness of home-based HIV counselling and testing in reducing stigma associated with HIV and risky sexual behavior among adults and adolescents. Participants were 13 and older. Outcomes of interest were clinical HIV outcomes, violence, stigma, and sexual behaviors.	yes	Meta-Analysis	9	The risk of observing or experiencing stigmatizing behavior was lower among the intervention population. The risk of IPV was also lower among those who participated in the program. Risky sexual behaviors like having multiple partners and/or casual sex partners also improved post-intervention.	High
Finnie, et al 2022	This review examined the effectiveness of interventions designed for primary prevention of IPV and sexual violence among youth aged 12–24. Outcomes of interest were perpetration, victimization, and bystander action. All included studies were conducted in high income countries.	no	Systematic Review	28	Review found evidence that suggested primary intervention programs were effective in reducing the perpetration of IPV and sexual violence among youth. Bystander action was found to improve in the short term. No consistent results found for incidence of victimization.	Critically low
Fox, et al 2016	This study reviewed evidence to identify what works to reduce victimization. Key outcomes were reduced victimization, enhanced beliefs/attitudes about victims, and, improved knowledge of victimization issues. Victimization was split into bullying, IPV, sexual assault, and general victimization.	no	Systematic Review	83	Some programs were effective at reducing bullying victimization but none of the IPV, sexual assault, or general victimization programs were found reduce incidences of victimization. Various school-based programs were effective at improving beliefs regarding victimization. Other programs effectively increased knowledge and awareness of victimization.	Moderate
Fulu, et al 2014	This review focused on interventions designed prevent violence or further violence against women and girls. Key outcomes were related to IPV, non-partnered sexual violence, and child abuse. Studies conducted in various settings including humanitarian and conflict-affected contexts.	yes	Non-Systematic Review	244	Concluded that there is enough evidence to recommend the following interventions: relationship-level, micro-finance combined with gender-transformative components, community mobilization targeting social norms, education and community programs targeting boys and men, and parenting programs. Insufficient evidence found for single focus communication campaigns.	NA
Gannon, et al 2019	The aim of this review and meta-analysis was to evaluate the success of offense specific psychological treatments provided to adjudicated perpetrators of violence. Recidivism was measured using convictions, arrests or charges, institutional records, unofficial and self-reports. Outcomes were general recidivism or recidivism of sexual, domestic, or general	no	Meta-Analysis	70	Evidence suggested that traditional and specialized psychological programs affect multiple offending behaviors and contributed to substantial reductions in offense specific recidivism. Program staffing found to be a critical moderating variable.	Critically low
Garner, et al 2020	This analysis examined the deterrent effects of criminal sanctions on IPV. The sanctions considered were prosecution, conviction, and incarceration. All cases analyzed took place in the United States or Canada.	no	Meta-Analysis	57	Analysis yielded mixed results. Slight deterrent effect associated with prosecution. No deterrent effect for conviction. Large escalation effect among incarcerated perpetrators.	Moderate
Gibbs, et al 2017	This review synthesized evidence on economic interventions designed to prevent HIV risk behaviors and IPV. Child and adult outcomes were evaluated. Interventions were categorized into cash transfers, economic strengthening, and economic strengthening and gender transformative.	yes	Non-Systematic Review	45	Cash transfer programs had neutral or positive outcomes. Economic strengthening interventions had mixed effects - null, negative, and positive. Interventions combining economic strengthening and gender transformative components more likely to have positive results.	NA
Gichuru, et al 2019	This review and analysis examined the impact of microfinance interventions on contraceptive use, childhood nutrition, and female empowerment. The review focused on South Asia, Sub-Saharan Africa, Latin America, and the Caribbean. Various types of microfinance programs included.	yes	Meta-Analysis	23	Analysis indicated microfinance programs led to increase in the number of women using contraceptives. Mixed results found regarding the impact of microfinance programs on IPV but there were some positive changes associated with female empowerment. Two studies reported improvements in nutrition.	High
Gilani, et al 2020	This review focused on programs designed to prevent sexual abuse among adolescents aged 10-20. Populations included males and females. Key outcomes were incidence of sexual assault and harassment and awareness and attitudes related to sexual violence.	yes	Systematic Review	10	Adolescent empowerment and self-defense interventions were associated with significantly lower prevalence of sexual harassment and assault. A statistically significant correlation was found between adolescents' increased knowledge and attitudes and the reduction of risk factors and instances of sexual assault. Promotion of awareness and attitudes and education programs	Moderate

Author year	Aims and Population	Includes LMICS	Type of Review	# of studies	Results	Quality Score
Gilbert, et al 2015	This review synthesized evidence that explored the link between epidemics of substance abuse, violence and HIV/AIDS. It also sought to identify interventions that targeted key mechanisms of said epidemics.	yes	Systematic Review	75	Robust evidence suggested that GBV significantly increases the likelihood of HIV and other STIs among women and girls who use drugs. Several behavioral, structural, and biological mechanisms link GBV and HIV. Identified GBV prevention and treatment interventions that can be merged with HIV prevention, testing, and treatment interventions to target these mechanisms among	Moderate
Gilchrist, et al 2015	This review evaluated IPV interventions that included CBT and anger management for adult male IPV offenders. The primary outcome was IPV perpetration. All trials were conducted in the United States.	no	Systematic review	6	Some trials had null effects and some had positive impacts. Overall, there was insufficient evidence to establish the effectiveness of interventions that combine CBT and anger management components for male alcohol abusers in reducing IPV.	Moderate
Giusto, et al 2018	This review included literature on interventions targeting men's alcohol use and any family related outcomes in LMICs. Studies could not include only females and participants had to be between 18-65. Family outcomes included parenting, IPV, communication, and family functioning.	yes	Systematic Review	9	Review found gap in evidence of interventions targeting men's drinking and its impact on families. Despite this, the majority of studies reviewed found modest improvements on drinking, family, and couples outcomes. Gender transformative approaches were linked to reduced IPV.	High
Goldfarb, et al 2021	The goal of this review was to determine the effectiveness of comprehensive sex education efforts. Outcomes included appreciation of sexual diversity, dating and IPV prevention, development of healthy relationships, prevention of child sex abuse, improved social/ emotional learning, and increased media literacy. All programs were conducted in the	yes	Systematic Review	80	Strong support found for comprehensive sex education across grades and topics. Evidence found substantiates effectiveness of programs that have an inclusive definition of sexual health and have an affirming approach to sexuality. Evidence indicated programs that begin sooner and are of longer duration are favorable.	Moderate
Graham, et al 2021	This review evaluated sexual and domestic violence and IPV prevention programs for boys and men. Programs across developmental periods and settings were included but they had to report on changes in perpetration.	yes	Systematic Review	10	Evidence was insufficient and no conclusions regarding programs' effectiveness could be drawn. Significant heterogeneity found across programs' approaches, designs, measurements, and findings.	High
Hackett, et al 2016	The goal of this study was to analyze the effectiveness of domestic violence mental health interventions targeting victims of IPV and child witnesses. Outcomes were split into external stress, psychological adjustment, self-concept, social adjustment, family relations, and maltreatment occurrences.	no	Meta-Analysis	17	Medium to large effect size associated with interventions across outcomes for both internalized and externalized indicators. Research related to maltreatment events and self-concept had inconsistent results. Evidence reviewed supports notion that IPV interventions have mutually beneficial effects for adults and children.	Critically low
Hameed, et al 2020	This review's objective was to assess the effectiveness of psychological therapy programs designed for women who experienced IPV. Primary outcomes were depression, self-efficacy and an indicator of harm. Secondary outcomes included other mental health symptoms, anxiety, quality of life, re-exposure to IPV, safety planning and behaviours, use	yes	Meta-Analysis	33	Analysis indicated that therapy programs can reduce depression and anxiety. Data was inconclusive regarding efficacy, post-traumatic stress disorder, re-exposure to IPV, and safety planning. Insufficient data on harm.	High
Hardee, et al 2014	This paper synthesized evidence regarding social and structural drivers of HIV for women: gender norms; violence against women; legal norms to empower women; women's employment, income and livelihood; education for girls and stigma and discrimination. It then evaluated the success of structural interventions designed to strengthen the 'enabling	yes	Systematic Review	64	Results showed that interventions targeting key social and structural drivers led to increasing HIV-protective behaviors and reduced risk behaviors, improving services, and widows' ability to cope with HIV. Interventions were also effective at making relationships more gender-equitable and decreasing violence. These positive effects were particularly effective among young people.	Critically low
Heard, et al 2020	This study reviewed applied theater interventions devised to address primary, secondary, and tertiary IPV prevention. Interventions were conducted with varied groups such as young people, survivors, service providers, and university students. Interventions were conducted in the United States, United Kingdom, or Australia.	no	Systematic Review	16	At a primary level, interventions were found to be successful at creating awareness of IPV and being a useful tool for healthy relationship modelling with young people. Evidence also indicated that these interventions may enhance skills and contribute to healing for IPV survivors. Interactive and participatory methods associated with larger impacts.	Moderate
Hollander, et al 2018	This report reviewed the evidence on women's self-defense training programs and their outcomes. Also investigated whether self-defense training reduced women's risk of violence. Critiques, possible harms, and the impact on different groups of women were considered as well.	yes	Non-Systematic Review	6	Found that empowerment-based self-defense training can reduce the likelihood of attempted assaults. Other positive effects identified were increased self-confidence, less fear, and less feelings of blame.	NA
Hoppe, et al 2020	This meta analysis sought to determine whether mandatory arrests for domestic violence had an effect on repeat offending. All included studies were conducted in the United States or Canada.	no	Systematic Review	11	Results indicated that arrest did not significantly reduce likelihood of subsequent domestic violence perpetration and was not likely to have a deterrent impact on offenders.	Critically low
Howell, et al 2017	This review presented evidence on IPV's effect on pregnancy and examined evidence on interventions for IPV-exposed pregnant women. Interventions targeted reducing IPV victimization, mental health consequences of IPV, integrated (reducing IPV/mental health), and intergenerational risk (i.e. birth outcomes.)	no	Systematic Review	17	No interventions designed exclusively for women in the prenatal period have been successful at addressing the needs of IPV-exposed pregnant women (mental health, victimization, parenting.) Data regarding program effectiveness was insufficient and percursory.	Low
Hudspith, et al 2021	The goal of this review was to synthesize studies that evaluated interventions aimed at reducing rape myth acceptance. All included studies were conducted in North America, some studies included men and women. Common interventions were bystander training programs, gender role programs, and programs focused on risk reduction.	no	Systematic Review	20	Results indicated that interventions targeting rape myth acceptance can have a positive, albeit short-term, effect on participants. Effective programs included those that presented information about the rape myth, contained an empathy component, and those focused on bystander intervention. Programs found to be most effective were shorter and presented via video.	Critically low
Jahanfar, et al 2014	This review examined the effectiveness and safety of domestic violence prevention interventions targeting pregnant women. Primary outcomes were reduction of episodes of physical, sexual, and/or psychological violence and prevention of violence for up to a year post-birth.	no	Systematic Review	10	Unable to properly assess the effectiveness of the interventions due to insufficient evidence. Most studies failed to report whether there had been changes in incidences of violence.	High
Jewkes, et al 2015	This paper examined the effectiveness of VAWG interventions in preventing VAWG or addressing related risk factors. VAWG outcomes evaluated included child abuse or maltreatment, child sexual abuse, IPV, partner or non-partner sexual violence. Variety of intervention types and settings included.	yes	Non-Systematic Review	38	Protection orders and shelters found promising while perpetrator programs, advocacy interventions, arrest policies, and second responder programs yielded conflicting evidence. Routine screenings in health services and mandatory reporting/arrests were ineffective. Insufficient evidence for various other types of interventions.	NA
Jewkes, et al 2019	This report examined evidence generated by interventions to reduce VAWG. The interventions included were evaluated as part of the *What Works 1* program. Outcomes of interest included gender attitudes, roles, and social norms.	yes	Non-Systematic Review	5	to/over 50% reduction of violence within a programmatic cycle. Factors that influenced effectiveness were identified as context, a comprehensive theoretical approach to intervention design, appropriate program duration and coverage, establishing 'essential elements' of successful norm based interventions, integrate support for survivors along	NA

Author year	Aims and Population	Includes LMICS	Type of Review	# of studies	Results	Quality Score
Jonker, et al 2015	This review conducted a meta-analysis of studies evaluating interventions administered to female IPV victims during/after their stay in a women's shelter. Key outcomes were related to mental health, re-victimization, and social outcomes. Every study except one was conducted in the United States.	no	Meta-Analysis	10	Analysis yielded a significant overall effect for mental health outcomes, abuse outcomes, and social outcomes. The evidence suggested that interventions provided during and after women's stay at a shelter were effective at improving mental health, reducing re-abuse, and improving social outcomes.	Critically low
Jouriles, et al 2018	The goal of this review and meta-analysis was to evaluate bystander programs addressing sexual violence on college campuses. Outcomes assessed included participant's beliefs, attitudes, and bystander behavior. Included interventions had mixed and single sex groups.	no	Meta-Analysis	24	Analysis suggested that students who received the intervention had more pro-social attitudes and beliefs related to sexual violence and engaged in more bystander behavior. Significant changes lasted for at least three months before diminishing. Longer programs were more effective at improving attitudes and beliefs.	Moderate
Karakurt, et al 2016	This review and meta-analysis investigated the effectiveness of couples therapy interventions as a treatment of violence. All interventions were conducted in the United States. Comparison groups included no treatment controls, gender-specific individual therapy, and gender-specific group therapy.	yes	Meta-Analysis	6	Analysis results indicated that couples therapy significantly reduced IPV. Benefits of couples therapy can variable across couples and sample used was not very diverse.	Critically low
Karakurt, et al 2019	This study's aim was to assess the success of batterer intervention programs in reducing IPV for male offenders. The majority of the interventions were group-therapy based and were conducted in the United States.	yes	Meta-Analysis	13	Pooled results suggested that batterer interventions were successful at reducing violence for male IPV perpetrators. Interventions that addressed trauma and substance use tended to be more effective. Intervention programs with a gender-role focus had mixed results.	High
Katz, et al 2013	This meta-analysis evaluated the efficacy of bystander education programs on preventing sexual assault in college settings. All programs analyzed were conducted in North America.	no	Meta-Analysis	12	Results suggested moderate effects for bystander efficacy and intentions to intervene. Smaller but significant effects were found for self-reported helping behaviors, lower rape-supportive attitudes, and lower rape proclivity. The same was not true of perpetration.	Critically low
Keith, et al 2022	This study reviewed evaluations of VAWG interventions in Sub-Saharan Africa. Outcomes of interest were reported GBV occurrence, GBV-related norms, attitudes, and symptoms. Interventions were social, economic, socio-economic, and psychological empowerment programs.	yes	Systematic Review	53	Social empowerment interventions found to be effective at transforming attitudes and norms and reducing GBV. Psychological interventions had a positive impact on GBV related symptoms. Evidence related to economic empowerment programs was ambiguous.	Moderate
Kennedy, et al 2014	This review examined the evidence of income generation interventions that target HIV prevention in LMICs. Interventions including microfinance and vocational skills training were evaluated. IPV was a secondary outcome.	yes	Systematic Review	12	One study suggested income generation interventions had a positive effect on IPV reduction and several studies led to improvements in financial conditions. Majority of studies did not show a significant HIV-related behavioral outcomes.	Moderate
Kerr-Willson, et al 2019	This review presented evidence on interventions designed to prevent women's victimization and men's perpetration of physical/sexual IPV and non-partner sexual violence. Child and peer youth violence was also included. No limits on geographic scope or participants' age range.	yes	Systematic Review	96	Economic transfer programs, socio-economic empowerment programs targeting women, community and school-based interventions, interventions that address alcohol/substance abuse, interventions targeting female sex workers, and some couple's interventions found effective when well designed. Several other interventions were associated with conflicting, insufficient,	Moderate
Kettrey, et al 2019	This review evaluated studies that assessed the effects of bystander programs on bystander efficacy, intentions, or intervention among college students in the United States.	no	Meta-Analysis	14	Effects on bystander intentions were significantly stronger among students in their first two years compared to those in their later years of college. There was no evidence of a significant difference in effects on bystander efficacy or intervention.	Critically low
Kettrey, et al 2019	Studies synthesized evaluated bystander programs' effects on bystander intervention and self-reported perpetration of sexual assault among adolescents and college students. All studies evaluated took place in educational settings in the United States.	yes	Meta analysis	21	Results suggested that bystander intervention programs had a significant positive effect on bystander intervention but no significant impact on sexual assault perpetration. Evidence did not suggest that a gendered approach to program implementation makes a difference. Gendered framing or portrayal of sexual assault also had no effect.	Critically low
Kettrey, et al 2020	This systematic review and meta analyses examined bystander sexual assault prevention programs on bystander intervention prerequisites and or bystander intervention behavior among high schoolers and college students. To be included, studies had to take place in an educational setting. All included studies took place in the US except for one in India.	yes	Systematic Review	19	Bystander sexual assault programs had non-significant effect on participant's ability to notice or identify signs of sexual assault or effects on taking responsibilities and knowing appropriate intervention strategies. Analyses showed significant positive effect in regards to identifying appropriate situations for intervention but these effects were not long lasting. Results also	High
Kiani, et al 2021	This review examined the impact of empowerment intervention programs that aim to prevent domestic violence. The group of interest was women and girls who experience domestic violence - there was no geographic focus. Interventions included conditional cash transfers, motivational interviewing, among others.	yes	Systematic review	11	Interventions that included an economic component and those that are communication based found to be more effective at reducing domestic violence. Community based interventions did not have significant effects.	High
Kim, et al 2016	The goal of this meta-analysis was to update the the evidence base regarding the effectiveness of sex offender treatment. Effectiveness was measured by rate of recidivism. Included interventions targeted adolescent and adult sex offenders.	no	Meta-analysis	11	Analysis found that interventions contributed to recidivism. Interventions were more effective at reducing recidivism with adolescents. Chemical and psychological treatments found to be more effective than psychological ones.	Low
Kirk-Provencher, et al 2021	The aim of this review was to fill the evidence gap regarding the inclusion and representation of sexual and gender minority groups in bystander intervention groups on college campuses. Interventions had to seek to reduce dating and/or sexual violence and measure beliefs, attitudes, or bystander behaviors.	no	Systematic Review	28	Inclusion of sex and gender minorities is not frequently addressed in studies evaluating the efficacy of violence prevention bystander interventions. Most bystander intervention studies don't clarify whether they address sexual and gender minority students. Unclear whether these programs help sexual and gender minority students or address the risk factors they face.	Critically low
Kirk, et al 2017	This review focused on secondary and tertiary prevention interventions for VAW in LMICs. Included interventions addressed the needs of VAW survivors and aimed to prevent reoccurrence.	yes	Systematic Review	22	Weak and limited evidence did not allow for a definitive conclusion on what programs were effective. Some evidence found for the success of interventions focusing on alcohol use on secondary prevention and psychotherapy intervention effects on tertiary prevention. Evidence found for crisis center's increasing survivor's access to services.	High
Klein, et al 2019	This review focused on housing interventions for IPV survivors - what they are, their strengths and weaknesses, and their effectiveness. Outcome categories included IPV revictimization, housing stability, relationship, services, and mental health. All interventions evaluated were based in the United States.	no	Systematic Review	12	Limited evidence available on the effectiveness of shelters. Rapid rehousing and flexible funding seem to contribute to housing stability.	Moderate

Author year	Aims and Population	Includes LMICS	Type of Review	# of studies	Results	Quality Score
Langstrom, et al 2013	This review aimed to evaluate the effectiveness of medical and psychological interventions targeting individuals at risk of sexually abusing children - including known abusers. Primary outcomes included arrests, convictions, breaches of conditions, and self reported sexual abuse of children after at least one year.	yes	Systematic Review	8	Insufficient evidence regarding effectiveness of interventions for adults. For adolescents, some evidence suggested multisystemic therapy can prevent re offence. No eligible research found on preventative interventions targeting those who had not sexually abused before but were considered higher risk.	High
Leigh, et al 2022	This study synthesized the literature on the psychological treatments administered in the United Kingdom to address stalking. A variety of therapy modalities were reviewed. IPV was considered an associated offense.	no	Non-Systematic Review	9	Cognitive analytic therapy, cognitive behavioral therapy, and dialectical behavior therapy showed promising results. Multi-dimensional approaches seem to be more effective. Evidence base remains severely limited.	NA
Leite, et al 2019	This study's objective was to assess the impact of economic empowerment programs on the risk of sexual, psychological, or physical violence. The study focused on microcredit and cash transfer programs. Most studies focused on married or previously married women.	yes	Systematic Review	32	Evidence yielded mixed results regarding the effectiveness of microcredit programs on physical/sexual violence. Cash transfer programs had heterogeneous results. Economically empowered and more autonomous women could face increased risk of violence.	Moderate
Lester, et al 2017	This review of review focused on school violence prevention approaches. Outcomes were peer violence, corporal punishment, student-on-teacher violence, and teacher-on-student violence. Most of the research was conducted in North America. Interventions could include pre-primary, primary, and secondary students.	yes	Systematic Review	36	Cognitive behavioral and school-wide programs showed potential for reducing peer victimization. Evidence on peer mentoring/mediation, cognitive behavioral, and social-emotional programs suggested they were effective at preventing perpetration of peer violence. Insufficient evidence on student/teacher violence interventions.	High
Lilley-Walker, et al 2018	This review reported on the evidence relating to European domestic violence perpetrator programs. There were no limitations imposed on study design, measures, or outcomes.	no	Systematic Review	60	Further research needed to establish which domestic violence perpetrator programs are the most effective. Standardizing studies would facilitate comparisons.	Moderate
Linde, et al 2020	This review examined the effect of eHealth interventions compared with traditional approaches on reducing IPV, depression, and PTSD among women exposed to IPV. All types of eHealth interventions were included. Most trials were administered in the United States.	no	Meta-Analysis	14	Pooled analyses found no significant effect of eHealth interventions on any of the outcomes.	High
Lopez, et al 2021	This review evaluated the outcomes of sexual abuse prevention programs for people with developmental and intellectual disabilities. Most programs evaluated focused on acquiring behavioral abilities and sexual education. Programs included women-only and mixed gender samples.	no	Systematic Review	10	Some evidence pointed to the effectiveness of programs that focus on behavioral abilities. Majority of studies had insufficient evidence which inhibited proper evaluation.	Critically low
Lorenzetti, et al 2017	This review synthesized the evidence on integrated health and microfinance interventions. Most interventions examined combined microfinance and health education. A variety of health education and promotion related outcomes, which included IPV, were investigated.	yes	Systematic Review	35	Interventions that incorporated micro-loans and comprehensive training and education on IPV and HIV contributed to a reduction in IPV. Most interventions were associated with positive effects on health knowledge and behaviors but not health status. Multi-component interventions were generally effective but more evidence needed.	Low
Lundgren, et al 2015	The aim of this review was to identify effective strategies to prevent IPV and sexual violence among adolescents aged 10-19. Interventions covered targeted violence perpetrated by men against women in heterosexual couples. Outcome categories included behaviors, attitudes/skills, and knowledge.	yes	Systematic Review	61	School-based dating violence interventions were found successful. Evidence on community-based gender transformative interventions suggested they were effective at preventing IPV and sexual violence. Parenting interventions found to prevent risk factors like child maltreatment. But all successful interventions were conducted in high income countries.	Low
Malhotra, et al 2015	The goal of this review was to examine teen dating violence prevention research and identify gaps in the evidence with regard to interventions targeting Hispanic adolescents. Included studies were school or community based. Most studies had majority sample of White adolescents.	no	Systematic Review	18	More research needed that investigates the impact of violence prevention interventions on Hispanic folks and other groups. Programs that had positive effects on knowledge and attitudes related to teen dating violence had no lasting effect on violence victimization or perpetration. Better research design needed to investigate the relationship between theoretical concepts (i.e.	Low
Marcus, et al 2021	This review investigated evidence on parenting programs designed to change norms related to violence against adolescents in LMICs. Programs evaluated sought to reduce neglect, child marriage, and physical, sexual, or emotional violence against adolescents.	yes	Systematic Review	58	Evidence suggested that parenting programs were successful at reducing physical and emotional violence and neglect of adolescents. Programs also appeared to increase parents' ability to safeguard children from sexual violence and reduced their support for child marriage. Longer programs tended to be more effective.	Low
Marshall, et al 2018	Studies reviewed addressed IPV and HIV amongst adult women. The primary outcome for IPV included sexual, physical, and psychological violence; primary outcomes for HIV included condom use, number of sex partners, and incidence of HIV/ STI. There was no geographic focus.	yes	Systematic Review	14	Combined interventions that contained multiple strategies with multi-pronged, multi-layered approaches found to be the most successful at addressing both HIV and IPV. Need for multi-sectoral, system levels approaches. Empowerment, feminist, and gender theories proved important role in effective interventions.	Low
Matjasko, et al 2020	This review synthesized evaluations of microfinance interventions' impact on violence outcomes (intimate partner violence, sexual violence, physical violence, and child abuse and neglect.) Studies examined only included women and took place across Africa and South Asia.	yes	Systematic Review	14	Microfinance interventions can prevent and reduce violence among women, especially when programs include a gender equity or social support component. Some findings had null or negative impacts such as increased marital control, increased frequency and severity of violence, among others.	Low
McCloskey, et al 2016	This study synthesized the evidence on IPV intervention programs conducted across Sub-Saharan Africa. Most programs focused on violence prevention and reduction of HIV.	yes	Systematic Review	7	Some programs seemed to be successful at reducing violence perpetration among men. Risk factors found to be associated with violence included poverty, drinking, low education, previous experience of child abuse, PTSD, and traditional gender role beliefs. IPV prevention programs often combined with HIV programs.	Low
McNaughton, et al 2021	Studies reviewed investigated the effect of adolescent dating violence prevention programs among youths aged 10-19. Perpetration and victimization outcomes included included physical, sexual, and/or psychological violence. Most studies evaluated were conducted in high income countries.	yes	Systematic Review	52	Evaluations in high income countries more likely to measure effects on violence perpetration and victimization, also more likely to include boys and girls. Half of evaluations analyzed found to have a significant preventative effect on at least one outcome.	High
Meinck, et al 2019	This study explored the effectiveness of GBV interventions targeting young people aged 10-24 living with or affected by HIV in LMICs. Outcomes of interest were incidences of GBV or GBV related attitudes.	yes	Meta-Analysis	18	Sexual health and social empowerment interventions, sexual health and social empowerment programs combined with economic strengthening, self defense, and gender sensitization interventions can be effective for GBV exposure and improving GBV-related attitudes. The same does not apply for GBV perpetration. Safer school interventions showed no effects.	High

Author year	Aims and Population	Includes LMICS	Type of Review	# of studies	Results	Quality Score
Mendelson, et al 2015	This paper reviewed parent-focused prevention approaches of child sexual abuse. Programs reviewed were implemented in the United States.	no	Non-Systematic Review	12	Parent-focused prevention approaches are a promising approach to child sexual abuse prevention. There is potential to integrate parent-focused interventions with school, community, and societal-focused interventions.	NA
Mujal, et al 2021	This review included studies that evaluated sexual violence bystander intervention programs. Included studies took place in the United States or Canada. Most of the studies were conducted with college students.	no	Systematic Review	44	Sexual assault prevention programs found to be effective at improving attitudes and behaviors. Knowledge about said effectiveness still small due to a lack of rigorous evidence. Training approaches and outcome tools can translated to other violence prevention efforts.	High
Nesset, et al 2019	The aim of this review was to update the evidence on the effectiveness of CBT group therapy for male perpetrators of IPV. Interventions included targeted adult males aged 18 or older with a history of physical, psychological, or sexual violence towards their female partners. Outcomes of interest were reduction in violent behavior, physical and mental	no	Systematic Review	6	Evidence regarding the effectiveness of CBT group programs was inconclusive. Some trials found a reduction in IPV after treatment but studies were small and mostly relied on perpetrators' self reports.	HIgh
Neville, et al 2014	The goal of this review was to synthesize evaluations of brief violence interventions targeting male participants to inform the design of a Brief Violence Intervention strategy in Scotland. Restrictions regarding participant age, intervention location, and intervention content were not included in the search.	no	Rapid Review	10	Evaluations reviewed indicate brief violence interventions can be effective at reducing violence - both at the behavioral and cognitive level. Successful interventions included motivational interviewing, social norms approaches, working with victims in medical settings, discussion around alcohol use, computer-therapist hybrid delivery, and appropriate follow up.	NA
Newlands, et al 2016	This review examined sexual violence interventions implemented in United States college campuses. Programs were evaluated according to a prespecified set of criteria.	no	Non-Systematic Review	28	Separate gender programs were found to be more effective. Programs focusing on alcohol use and self-defense had the most promise for women. As for men, programs that addressed consent tended to be more impactful.	NA
NICE, et al 2013	This broad review looked at interventions designed to identify, prevent, reduce and respond to domestic violence between family members or between current or former intimate partners. Included studies were conducted in healthcare, social care, and specialized domestic violence settings. Populations of interest included children,	no	Systematic Review	899	Most prevention approaches targeting young people were secondary (rather than primary) prevention, and most focused on changing attitudes rather than reducing violence. Only weak evidence exists for interventions in healthcare settings, and interventions and prevention interventions with women at high risk of violence.	High
Nikolova, et al 2018	Studies included evaluated gender-informed interventions in Sub-Saharan Africa with an HIV-related outcome. Outcomes of interest included HIV behavioral outcomes, HIV, and measures of violence against women. Participants included adult males and females.	yes	Systematic Review	17	Most interventions had positive effects with longer interventions being more effective on average. Cultural and behavioral practices must be targeted with a community-based dynamic approach. Studies that address violence against women in relation to HIV must be further developed.	Critically low
O'Connor, et al 2021	This review examined Men's Behavior Change Programs. These programs sought to change perpetrators' violent attitudes and beliefs by making them accountable for their actions.	yes	Systematic Review	13	Despite limited evidence, positive impacts on multiple outcomes were found: communication, parenting, interpersonal relationships, aggression, abuse, responsibility for behavior, self-awareness power and control tactics, empathy, skills development, among others. Evaluations did not investigate the link between men's responsibility and the safety of women and children.	Moderate
O'Malley, et al 2017	This review synthesized the findings reported on the impact of microfinance interventions on women's health. Health outcome categories were HIV/AIDs, reproductive health, mental health, and violence.	yes	Systematic Review	41	Evidence was weak but indicated that microfinance interventions can have a positive effect across a range of women's health knowledge, attitudes, and outcomes. Insufficient evidence to draw a definitive conclusion regarding the impact of these programs.	Critically low
O'Doherty, et al 2015	This review evaluated the effectiveness of screening for IPV conducted in healthcare settings. The goal was to determine whether or not these screenings increase identification and referral to support agencies, improves women's wellbeing, reduces violence, or causes harm. Studies reviewed included women aged 16 and older.	no	Systematic Review	11	Evidence found that screening increased identification of IPV but no evidence to suggest that it increased referrals to support services. It was seldom reported and no evidence was found regarding reduction in IPV. A single study reported that there was no evidence of harm caused.	Moderate
Ogunjimi 2017	This review evaluated school-based education programs designed to prevent child sexual abuse. There was an emphasis on programs that included children, adolescents, teachers, and parents.	yes	Systematic Review	18	Evidence suggested that involvement of parents and teachers in early detection in child and adolescent abuse was crucial. Some evidence found to suggest an improvement in participants' communications and self-esteem. More evidence, especially in LMICs, needed.	Low
Orchowski, et al 2018	This study investigated sexual assault prevention programs conducted within military settings in the United States. Most participants were unmarried, new recruits, lived in military housing, and aged between 18-26.	no	Systematic Review	6	Most programs evaluated did not measure whether program participation affected rates of sexual abuse. Studies did not include a long-term follow-up period. Programs evaluated in the studies selected did not reflect programs that were being implemented in military settings.	Critically low
OPRE, et al 2018	This study summarized the evidence base on IPV findings related to home visiting models. Programs of interest were limited to those geared towards pregnant women, expectant fathers, and parents with children in kindergarten or bellow.	no	Non-Systematic Review	16	Evidence indicated that Healthy Families America and the Nurse-Family partnership had a significant effect on IPV outcomes. Favorable effects measured included maternal perpetration, partner incidents resulting in injury, partner physical abuse, and maternal exposure to domestic violence.	NA
Orton, et al 2016	This review assessed the impact of group-based microfinance programs that are based on collective empowerment on health-related outcomes. All interventions evaluated targeted poor women living in LMICs.	yes	Systematic Review	23	Results indicated an association between program participation and health-related outcomes for women and children. Observed outcomes included reduced maternal and infant mortality, improved sexual health and, in some trials, reduction of IPV. Large and well-established programs were usually associated with increased empowerment which might have contributed to improved contraceptive use, well-being, and reductions in risk of IPV.	High
Parkes, et al 2016	This review examined research evidence on school related gender based violence. Despite its global scope, interventions in low and middle income countries were emphasized. School related GBV included physical, sexual, and psychological acts of violence.	yes	Non-Systematic Review	49	Most studies evaluated short programs with little or no follow up. Programs that don't target sexual violence specifically tend to be gender blind. Most effective interventions acknowledge the links between violence, identities, social norms, and intersecting structural dynamics.	NA

Author year	Aims and Population	Includes LMICS	Type of Review	# of studies	Results	Quality Score
Perez-Martinez, et al 2021	This study reviewed evaluation studies of educational interventions that addressed hegemonic masculinities and aimed to prevent GBV among adolescents. Hegemonic masculinities included gender roles, patriarchical norms, attitudes related to IPV, etc. Most studies were conducted in Africa.	yes	Systematic Review	15	Most studies showed reductions in physical and/or sexual IPV victimization and perpetration post-intervention. All of the interventions evaluated were associated with positive impacts on gender roles, GBV-related attitudes and myths.	High
Petering, et al 2014	This reviewed analyzed evaluations of IPV prevention programs with a focus on youth between the ages of 12 and 26. It aimed to compare results of IPV prevention among the general youth and at risk-populations and determine whether IPV programs are applicable to homeless youth.	no	Systematic Review	13	IPV prevention programs targeting youth have mixed effects. Most programs targeting at risk youth appear to still be in development. Whether existing programs can be adapted to homeless youth remains unclear, a new context-specific approach might be needed.	Low
Piolanti, et al 2022	This review examined the success of prevention programs for sexual and physical dating violence in adolescents. Study participants had to be 18 or younger. The majority of studies were conducted in schools in North America.	no	Meta-Analysis	18	Analysis found that interventions were significantly associated with reduced violence perpetration and survivorship. The reduction in sexual violence observed was not statistically significant. No significant relationship found between effectiveness and program length or intensity.	High
Prosman, et al 2015	The goal of this review was to evaluate the effectiveness of home visiting interventions on IPV experienced by mothers. The population of interest was abused mothers and abused children.	no	Systematic review	19	Programs that prioritized IPV and supported mothers showed statistically significant short term reductions in IPV. Insufficient evidence to determine the long-term impact of home visiting programs on IPV.	High
Ramsoomar, et al 2019	This report presented evidence that suggests there is an association between alcohol and substance use, mental health, and IPV. Interventions evaluated sought to tackle harmful alcohol and substance use. Outcomes evaluated centered around IPV and mental health.	yes	Non-Systematic Review	11	Evidence found indicates that preventing VAWG is associated with improved mental wellbeing and the same applies for peer-violence prevention and children. Couples therapy interventions that address alcohol and substance use and mental health challenges can reduce IPV experience and perpetration. Gender-transformative interventions can decrease harmful alcohol use and IPV	NA
Righi, et al 2019	This review included studies that evaluated integrated IPV and HIV interventions and targeted adolescents. Only interventions in Sub-Saharan Africa were included. Adolescents were between 13-18 years old.	yes	Systematic Review	6	Few HIV/IPV interventions specifically target adolescents. Mixed results found regarding reduction of sexual risk behavior and improvement of gender norms. Incosistent use of measurements and definitions of HIV and IPV found across studies.	Moderate
Rivas, et al 2015	The goal of this review was to assess the efficacy of advocacy interventions among women who experience IPV. Key outcomes were physical, sexual, and emotional abuse, depression, quality of life, and deaths. Studies involved participants aged 15-65.	yes	Meta-Analysis	13	Results suggested intensive advocacy can improve quality of life for women in shelters/refuges and can reduce physical violence up to 2 years post-intervention. No evidence was found that intensive advocacy reduced sexual or emotional violence, abuse, or that it contributed to imrpovements in women's mental health. Evidence on brief interventions is limited but might	High
Rivas, et al 2019	This review's objective was to investigate advocacy interventions for IPV, specifically what interventions worked and for whom, why they worked, and in what settings. Women who were 15 and older and had experienced or were experiencing IPV were the intended population.	no	Systematic Review	98	Evidence confirmed that core components of advocacy interventions such as education and information on abuse, rights and resources, referral and liaising with other services, risk assessment, and safety planning can be effective. Evidence suggested that if interventions are tailored to women's situations and admnistered for long enough they should benefit the participant in at least one outcome. In situations of severe abuse, advocacy intervention can	High
Robbers, et al 2017	This review explored approaches used to address sexual violence among refugee women in low-resource settings. Included studies addressed prevention and/or response to sexual violence. Evaluated trials included refugee women and girls or vulnerable women, healthcare providers, and displaced communities.	yes	Non-Systematic Review	29	Evidence indicated that programs that focus on engagement and participation as well as training and education can help address underlying causes of sexual violence. Interventions that involve community members, engage with harmful gender norms, and enable cooperation among stakeholders can help make the most of limited resources.	NA
Rose-Clarke, et al 2019	This review evaluated peer-facilitated community-based interventions for adolescent health in LMICs. The median age of adolescents was between 10-24. Outcome areas covered: infectious and vaccine preventable diseases, under-nutrition, HIV/AIDS, sexual and reproductive health, unintentional injuries, violence, physical disorders, mental	yes	Systematic Review	20	Limited evidence that suggested interventions improved mental health and reduced violence and substance use but not sufficient to make definitive conclusions. . No evidence of trials having positive effects on any of the other outcome categories.	High
Russell, et al 2021	The goal of this review was to synthesize all evidence on adolescent dating violence prevention programs that measured perpetration and/or victimization. All adolescents had to be 18 or younger at baseline. Outcomes were overall, emotional, physical and sexual adolescent dating violence or the threat of violence.	no	Meta-Analysis	10	Results suggested that adolescent dating violence programs signficantly reduced emotional and sexual adolescent dating violence among treatment groups. Results also showed that the risk of emotional and physical perpetration and victimization was lower for program partipants. No significant effects found for threatening or overall adolescent dating vilence perpetration or	Critically low
Sabri, et al 2019	This review synthesized and evaluated the efficacy of comprehensive, integrated, multi-component interventions for victimized substance-using men and women. Included interventions evaluated at least two syndemic outcomes: violence, HIV risk, substance misuse, and mental health. Most programs evaluated were admnistered in the United	yes	Systematic Review	7	Goal setting, psychoeducation, social support, empowerment strategies, risk assessments, safety planning and referrals to community resources were found to be effective components of interventions that addressed IPV. Other components that were specifically effective at tackling HIV risk, mental health outcomes, and substance misuse were also identified.	Critically low
Sabri, et al 2019	This review synthesized evidence on comprehensive multi-component interventions for victimized substance-using people. A multi-component intervention encompassed an intervention with combined components that addressed two or more syndemic outcomes; HIV risk, mental health, and substance misuse. Most studies were conducted in the United	no	Systematic Review	17	Components found to have a positive effect across multiple outcomes included skills training, psychoeducation, goal setting, empowerment strategies, risk assessment, among others. Stronger interventions and more rigorous evaluations needed. More programs tailored to the needs of diverse groups of victimized individuals who use substances needed.	Critically low
Salas, et al 2020	This review collected and evaluated evidence on IPV interventions. Prevention and treatment interventions were included. Populations varied across studies including women victims of IPV, male perpetrators, adolescents, adult couples, and specialist workers.	yes	Systematic Review	24	Results indicated that prevention interventions are effective. Follow-up on couple's interventions presented as their main limitation.	Moderate
Santirso, et al 2020	This review analyzed evidence on interventions for IPV offenders that included motivational strategies. Outcomes measured were reduction of physical and psychological IPV, rates of treatment dropouts, official recidivism of IPV offending, and intervention attendance dose. Program samples had to consist of adults and had to include men.	no	Meta-Analysis	12	Results indicated that IPV interventions that included motivational strategies were more effective at reducing dropout rates and increasing intervention dose. Evidence indicated that motivational interventions were more effective at reducing physical and psychological IPV and official recidivism but this was not a significant difference.	High
Sapkota, et al 2019	This aim of this review was to examine the approaches and effects of domestic violence interventions among pregnant women in LMICs. Outcomes included frequency and/or severity of domestic violence, mental health, safety behaviors, and use of community resources.	yes	Systematic Review	5	According to the evidence, supportive counselling interventions contributed to a reduction in domestic violence and an increase in safety behaviors. Limited evidence on effects on quality of life and use of community resources. Development of a theory of change regarded as a critical for domestic violence intervention development.	High

Author year	Aims and Population	Includes LMICS	Type of Review	# of studies	Results	Quality Score
Schmucker, et al 2015	This review was conducted to update the evidence base and evaluate the effects of sexual offender treatment on recidivism. Only official measures of recidivism were of interest. All included trials evaluated psychosocial programs - mainly CBT focused.	no	Meta-Analysis	29	Mean effect size for official recidivism was statistically significant. Programs that included cognitive-behavioral and multi-systemic treatment, small samples, medium- to high-risk offenders, more individualized treatment, and good descriptive validity tended to be more effective. Treatments admnistered in prisons yielded some positive outcomes but were not	Low
Semahegn, et al 2019	The goal of this review was to establish whether interventions focused on gender-norms are effective in preventing domestic violence against women in LLMICs. Target population had to be women aged 15-49. Domestic violence/IPV were assessed as lifetime and current.	yes	Meta-Analysis	52	Interventions that included legal framework or focused on gender norms found to be the most important in violence prevention. Community mobilization programs and awareness creation initiatives to change gender norms were associated with reducing IPV rates by half. Economic empowerment interventions contributed to reduced economic abuse but did not significantly	High
Shorey, et al 2014	The purpose of this review was to evaluate specific components of coordinated community responses for IPV victims. Components included advocacy research, criminal justice research, counselling, child services, healthcare, education, vocational, and media.	no	Non-Systematic Review	10	Some components, such as advocacy interventions, were identified at being effective in reducing women's risk of reabuse and improving their quality of life. Lack of empirical research evaluating other component's efficacy. Evidence indicated that integration of coordinated community responses might not be happening in several areas.	NA
Singh, et al 2018	The aim of this review was to assess the effect sexual and reproductive health interventions during humanitarian crises. Primary health outcomes evaluated were adolescent, maternal and neonatal morbidity; adolescent, maternal and neonatal mortality; STI diagnosis; GBV; and unmet need for family planning.	yes	Systematic Review	29	High quality evidence found to support the effectiveness of home visits and peer-led initiatives, training of lower-level healthcare providers, involvement of community health workers , having a network of health workers providing services, integration of HIV and sexual and reproductive health services, and men's discussion groups to reduce IPV. Moderate evidence found regarding	Moderate
Sinnott, et al 2016	This review examined evidence on IPV prevention approaches that target women during childbearing years. The focus was on childbearing years or prenatal period up to first five years post-birth. Most approaches identified were directed at mothers.	yes	Non-Systematic Review	43	Evidence base on prevention of female IPV victimization of women's medical care and home visitation programs, rendering mothers responsible for mitigating risks of violence to themselves and their children. IPV prevention programs for fathers still nascent. Promising evidence suggested that fatherhood motivated men to cease IPV perpetration.	NA
Small, et al 2013	This review investigated gender based HIV intervention research conducted in Sub-Saharan Africa. Study outcomes included biological outcomes, HIV risk, behavioral outcomes, violence, and risk reduction. Most studies had mixed male and female samples.	yes	Systematic Review	11	Half of the interventions reviewed were successful in increasing HIV knowledge and/or behaviors and improving attitudes towards women and gender roles. Role plays; microfinance and community engagement; HIV testing, counseling, and attitude change interventions were identified as promising. Longer interventions tended to be more effective.	Critically low
Spangaro, et al 2014	This review presented evidence on eleven different areas of intervention for health services responding to domestic violence. Several approaches were identified including routine screening; risk assessment and safety planning; first line responses; counselling with survivors; mother-child interventions; home visting and others. Program-specific	no	Non-Systematic Review	14	The review makes a wide range of recommendations for health systems based on existing evidence, including systematizing safety planning, prioritizing supportive advocacy in community health services, antenatal patients should be prioritized for counseling (and counseling should be administered by a social worker with specialist domestic violence training), and health services	NA
Spangaro, et al 2021	This review evaluated studies of interventions targeting sexual violence and IPV in conflict, post-conflict, and other humanitarian crises settings. Type and content of intervention included personnel, community mobilization, social norms, economic empowerment, empowerment, and survivor responses.	yes	Systematic review	18	Interventions that focus on social norms proved promising and worked well in conjuction to economic empowerment programs. Some evidence of reduced risk of sexual violence or IPV reported for all intervention types. Interventions with combined strategies prove effective.	High
Spencer, et al 2021	The meta analysis focused on studies that examined web based anger management programs or relationship education programs designed to reduce IPV. The outcomes of interest were anger, depression, emotional and physical IPV. No particular geographic or population focus.	no	Systematic Review	6	Results suggested that web based IPV/anger management programs had strong signficant effects in reducing depression and significant medium effects in reducing anger and physical and emotional IPV. Highlighted importance of online resources.	Low
Sprague, et al 2017	The goal of this review was to provide an overview of the evidence on IPV assistance programs within health care settings. Categories of assistance programs included counselling/advocacy, safety assessment/planning, referral, providing IPV resources, home visitation, case manage-ment, videos, provider signaling, and system changes.	yes	Systematic Review	43	Most programs covered were found to be beneficial to women. Programs led by a counsellor, community worker, or case manager and programs that consisted more than five sessions. No identified studies reported negative effectiveness or incidences of harm.	High
Stanley, et al 2015	The aim of this report was to summarize and evaluate the evidence on preventative domestic abuse interventions for young people under 18. Most interventions were admnistered in schools.	no	Non-Systematic Review	22	Stronger evidence found for interventions tackling changes in knowledge and attitudes than for interventions attempting to cause behavior change. Addressing social norms among peer groups and involving young people in the design and delivery of programs proved effective. Longer interventions delivered by trained leaders, namely teachers, proved more effective.	NA
Stark, et al 2021	This review evaluated the evidence on Women and Girls Safe Spaces programs designed to address VAWG in humanitarian contexts. All studies reviewed incorporated components from the safe space model, including life skills curricula, mentorship, and caregiver-targeted trainings or activities.	yes	Systematic review	7	None of the studies reported reductions in exposure to or rate of VAWG. Moderate improvements found in psychosocial well-being, social support, and attitudes regarding rites of passage.	Moderate
Stephens-Lewis, et al 2021	The goal of this analysis was to ascertain the effectiveness of interventions designed to reduce IPV among men who use substances. Studies had to include a heterosexual male population wherein at least 60% were alcohol and/or drug users. Most common intervention types were cognitive behavioral therapy and motivational interviewing.	yes	Meta-analysis	9	Results from individual trials showed a reduction in substance use in the short term and IPV pepetration at different time points. Meta-analysis with integrated substance use and IPV outcomes showed no signficant effects.	High
Stewart, et al 2021	This review examined interventions designed to address gendered stereotypes and norms across multiple outcomes of gender inequality. Populations were women and men/boys and girls of any age. Outcomes of interest included behavior, behavioral intentions, attitudes, and social norms.	yes	Systematic Review	30	Peer engagement, addressing multiple levels of the ecological framework, developing agents of change, modelling and co-design of interventions with participants or target populations all appeared to improve intervention impact. Cohort sex, program length, and follow-up data collection found capable of influencing program success. Interventions most successful in their	Moderate
Stith, et al 2022	This review sought to understand the effectiveness of systemic interventions in reducing IPV or child maltreatment. Systemic interventions included interventions that targeted couple or family interactions and/or processes. Intervention approaches included relationship education, "naturalistic" couple therapy, relational therapy, family therapy,	no	Systematic Review	9	Interventions were categorized according to the evidence gathered. Cognitive behavioral programs, parent education, and family therapy programs to reduce child maltreatment were labeled "possibly efficacious." Parent training programs that included live coaching of parent-child interactions were found to be "well-established." Programs based on naturalistic couples	Low
Storer, et al 2016	This review summarized the evidence on the impact of bystander interventions on participant's behaviors and attitudes related to sexual assault and/or dating violence. All programs targeted college students except for one with high school athletes. Main outcomes were use of bystander behaviors, willingness to intervene, confidence to	no	Non-Systematic Review	15	Evidence suggested that bystander programs can be effective at increasing participants' self-reported willingness and confidence to intervene - there is limited and mixed evidence to suggest that this translates to use of bystander behaviors. Insufficient evidence to determine whether programs contribute to a reduction in sexual assault/dating violence.	NA

Author year	Aims and Population	Includes LMICS	Type of Review	# of studies	Results	Quality Score
Tarzia, et al 2020	This review evaluated evidence on interventions for male perpetrators or victims of IPV in health settings. Key outcomes included male perpetration of IPV, alcohol/substance use, mental heatlh, and identification of perpetrators and/or referrals of perpetrators or victims.	yes	Systematic Review	14	Psychological therapies that addressed alcohol use were identified as the most promising intervention with male perpetrators. Remaining evidence on effectiveness of interventions was weak.	High
Tirado-Munoz, et al 2014	This systematic review and metanalysis reported on the effects of cognitive behavioral therapy and advocacy interventions on reducing IPV for survivors. Only data from RCTs was included, with populations of women ages 18+.	yes	Meta-Analysis	23	Both advocacy and CBT interventions reduced physical and psychological IPV, but not measures of sexual or any IPV.	High
Tol, et al 2019	This review reported on the IPV outcomes of mental health treatments in LMICs. Study populations included both IPV survivors and perpetrators, both male and female.	yes	Systematic Review	11	Two of the seven included studies (across 11 papers) evaluated interventions to reduce depression depression, and these interventions demonstrated an affect on short-term IPV. However more evidence is needed to asses whether mental health treatments may be an effective strategy to prevent or reduce IPV in LMICs.	High
Trabold, et al 2020	This review examined the efficacy and effectiveness of interventions for victims of IPV related to mental and physical health and revictimization. Studies included women aged 17 and older and most of the studies were conducted in the United States.	yes	Systematic Review	57	Interventions centered on empowerment-based advocacy and cognitive approaches had strongest positive outcomes. Other intervention approaches that proved to positively affect the physical and mental health of women who experience violence included problem solving, enhanced choice making, alteration in distorted self-thinking and perception.	Critically low
Travers, et al 2021	This systematic review and meta-analysis focused on interventions to prevent recidivism in IPV perpetrators. The review included any studies that involved IPV perpetrators ages 18+, but excluded studies where self-reported perpetration was the only outcome measured.	no	Meta-Analysis	31	Risk-need-responsivity interventions to prevent recidivism in IPV perpetrators demonstrated significant effects in the short- and medium- term, but failed to demonstrate significant effects at long-term follow-up.	Moderate
Turner, et al 2020	This meta-analysis evaluated evidence on psychosocial interventions for IPV in LMICs using data from RCTs. Included studies measured IPV as reported by female participants.	yes	Meta-Analysis	13	Psychosocial interventions included in this review demonstrated reductions across any form of IPV. Sexual IPV was reduced at the longest follow-up evaluation, but showed no significant effect at the shortest follow-up.	High
van Daalen, et al 2022	This mixed-methods systematic review assessed the impact of conditional and unconditional cash transfers on health outcomes and use of health services in humanitarian settings.	yes	Systematic Review	23	Violence was only measured in one of the 23 included studies, and the relationship between cash transfers and violence was not clear. More evidence is needed to understand the relationship between cash transfers and a range of health outcomes, in different humanitarian settings, over longer periods of time.	Moderate
Van Parys, et al 2014	This review is focused on interventions to prevent IPV around the time of pregnancy. Included studies evaluated interventions with pregnant women of any age and/or women who had given birth in the past year, using an RCT. The primary outcome of included studies had to be any measure of IPV.	yes	Systematic Review	9	There is a dearth of experimental evidence of interventions to prevent IPV around pregnancy. Some interventions did demonstrate promising effects on IPV, home visitation programs and some counseling interventions in particular.	High
Vargas, et al 2020	This review synthesized evidence regarding behavioral interventions to promote sexual and reproductive health among United States' military service members.	no	Systematic Review	15	Evaluations of interventions with the United States military population are limited. Only five of the included studies focused on sexual assault, and only two of these assessed behavioral outcomes. These interventions improved attitudes and self-efficacy to serve as a bystander.	Critically low
Walsh, et al 2015	This review sought to assess the effectiveness of school-based education programs focused on child sexual abuse among students aged 5-18. Outcomes of interest included students' protective behavior, knowledge of sexual abuse prevention, retention of protective behavior, retention of knowledge, harm (parental/child anxiety), and disclosure of sexual	yes	Meta-analysis	24	Studies reviewed indicated school-based programs had a positive impact on children's knowledge of sexual abuse and protective behavior and that this was consistent regardless of program type. Participation in the programs did not seem to affect feelings of fear or anxiety. Programs were associated with higher likelihood of sexual abuse disclosure but more research needed.	High
Wigham, et al 2022	The review focused on studies that evaluated recent interventions aimed at reducing violence for perpetrators. Most studies were conducted in the United States but had no exclusionary geographic criteria.	yes	Systematic Review	18	Studies that demonstrated statistically significant intervention effectiveness included motivational interviewing combined with cognitive behavioral therapy and continuing care, gratitude sharing and blessing counting, and online emotion regulation and conflict resolution.	Critically low
Williams, et al 2022	The main goal of this review was to identify women's economic empowerment interventions that measured IPV in South Asia and to synthesize those findings into the global evidence base. The review only included programs where women and girls were the sole recipients or main recipients of the economic empowerment efforts.	yes	Non-Systematic Review	30	Participating in a microfinancing program alone did not seem to reduce IPV. Cash and 'in-kind' transfers appeared to have mixed effects. Economic empowerment programs that targeted women's labor force participation did not show a signficant improvement on physical IPV.	NA
Wilson, et al 2014	This review examined studies that evaluated whether alcohol interventions/policies were associated with a reduction in IPV in adults. There was no geographic criteria. Interventions included community, relationship, and individual level programs.	yes	Systematic Review	11	Population pricing and taxation programs showed weak or no positive impact on IPV occurrence. Community level policies or interventions found weak evidence of association with IPV. Impact on IPV could not be attributed to couples interventions due to intervention design. Combined alcohol and violence treatment associated with some positive effects.	Low
Wilson, et al 2021	This review assessed the impact of post-arrest court mandated interventions for IPV offenders that targeted male offenders. All studies evaluated were conducted in North America except for one in Australia. Included studies had to include repeat IPV obtained at least 6 post-treatment.	no	Meta-analysis	21	Rates of lower IPV perpetration to those attending mandated interventions not statistically significant. Victim reported outcomes showed no signficant effect either way. Not enough evidence to establish the effectiveness nor potential harms of court mandated programs for IPV.	High
Wong, et al 2021	This review included studies that evaluated a college dating prevention programs or campaigns. The outcomes of interest were knowledge, attitudes, or bystander efficacy, intentions, or behavior. The vast majority of programs evaluated were conducted in the United States.	yes	Meta-analysis	29	Evidence suggested that programs were effective at improving knowledge, attitudes, and bystander skills. The same did not lead to increasing likelihood of bystander behaviors. Including content on types of violence in programs found to be a key component.	High
Wright, et al 2020	Studies included in this review had to evaluate a male-targeted prevention program intended to reduce negative attitudes, beliefs, and/or behaviors associated with sexual assault and rape. Included studies could not include female participants and all males were 18 or older. No geographic criteria was determined.	no	Meta-analysis	29	Male-targeted sexual assault prevention programs found to have a small positive impact on attitudes and intentions to engage in future sexually violent behavior. No evidence found to suggest these programs reduce occurrence of sexual assault.	Critically low

Author year	Aims and Population	Includes LMICS	Type of Review	# of studies	Results	Quality Score
Yanez-Peñúñuri, et al 2019	This study reviewed therapeutic interventions for victims and perpetrators of dating violence focusing on adolescents. Therapeutic interventions defined as having the aim to change habits, improve physical and mental wellbeing, and minimize distress. The studies took place in either Colombia, Mexico, Puerto Rico, or the USA.	yes	Systematic Review	10	Brief hospital-based therapeutic interventions can reduce victimization behaviors associated with dating violence but more evaluation is needed. The use of computers along with therapeutic practices can reduce victimization of physical violence but effect was not found for sexual violence. Physical violence found to be reduced in a study that employed a celphone application.	Low
Yount, et al 2017	This review synthesized reviews that evaluated interventions to prevent VAWG among girls/women aged 10-24 in LMICs. Outcomes of interest included child marriage, IPV, sexual violence, child abuse, and FGMC. Multiple types of interventions included.	yes	Systematic Review of Reviews	18	Interventions that encompassed community engagement, skill-building, and social network expansion showed potential to reduce VAWG. Interventions found to be less common in LMICs. Most interventions focused on secondary victimization prevention rather than on the prevention of perpretation.	NA
Zhang, et al 2021	This meta-analysis included evaluations of child sexual abuse (CSA) prevention or intervention education programs based in Chinese schools. Evaluations had to include a control or comparison group and provide sufficient data to calculate effect sizes. Evaluations included children who were under 18 years at the time of the program.	yes	Meta-analysis	7	School-based CSA prevention education programs demonstrated significant improvement in children's prevention knowledge and prevention skills, and these programs are more effective when instructed by researchers rather than teachers. Effects were greater in preschoolers as compared to elementary and middle-school students.	Moderate
