# Supplementary material for: Interventions to prevent violence against women and girls globally: a global systematic review of reviews to update the RESPECT women framework
Source: BMJ Public Health. 2025 Jan 20;3(1):e001126. doi: 10.1136/bmjph-2024-001126 (PMC11816861; doi:10.1136/bmjph-2024-001126)
Supplement: online supplemental file 4 [file bmjph-3-1-s004.pdf]

Akoensi, T. D., Koehler, J. A., Lösel, F., & Humphreys, D. K. (2013). Domestic Violence Perpetrator Programs in Europe, Part II: A Systematic Review of the State of Evidence. *International Journal of Offender Therapy and Comparative Criminology*, *57*(10), 1206–1225. https://doi.org/10.1177/0306624X12468110

Ali, P., Allmark, P., Booth, A., McGarry, J., Woods, H. B., & Seedat, F. (2021). How accurate and effective are screening tools and subsequent interventions for intimate partner violence in non-high-risk settings (IPV)? A rapid review. *Journal of Criminal Psychology*, *11*(4), 273–300. https://doi.org/10.1108/JCP-03-2021-0007

Alvarez, C. P., Davidson, P. M., Fleming, C., & Glass, N. E. (2016). Elements of Effective Interventions for Addressing Intimate Partner Violence in Latina Women: A Systematic Review. *PLoS ONE*, *11*(8), e0160518. https://doi.org/10.1371/journal.pone.0160518

Anderson, E. J., Krause, K. C., Meyer Krause, C., Welter, A., McClelland, D. J., Garcia, D. O., Ernst, K., Lopez, E. C., & Koss, M. P. (2021). Web-Based and mHealth Interventions for Intimate Partner Violence Victimization Prevention: A Systematic Review. *Trauma, Violence & Abuse*, *22*(4), 870–884. https://doi.org/10.1177/1524838019888889

Anderson, J. C., Campbell, J. C., & Farley, J. E. (2013). Interventions to Address HIV and Intimate Partner Violence in Sub-Saharan Africa: A Review of the Literature. *The Journal of the Association of Nurses in AIDS Care : JANAC*, *24*(4), 383–390. https://doi.org/10.1016/j.jana.2013.03.003

Arango, D. J., Morton, M., Gennari, F., Kiplesund, S., & Ellsberg, M. (2014). *Interventions to Prevent or Reduce Violence Against Women and Girls: A Systematic Review of Reviews*. http://hdl.handle.net/10986/21035

Araújo, G. E., Cruz, O. S., & Moreira, D. (2023). Maladaptive Beliefs of Young Adults in Interpersonal Relationships: A Systematic Literature Review. *Trauma, Violence & Abuse*, *24*(2), 646–661. https://doi.org/10.1177/15248380211038684

Arce, R., Arias, E., Novo, M., & Fariña, F. (2020). Are Interventions with Batterers Effective? A Meta-analytical Review. *Psychosocial Intervention*, *29*(3), 153–164. https://doi.org/10.5093/pi2020a11

Armenti, N. A., & Babcock, J. C. (2016). Conjoint treatment for intimate partner violence: A systematic review and implications. *Couple and Family Psychology: Research and Practice*, *5*(2), 109–123. https://doi.org/10.1037/cfp0000060

Arroyo, K., Lundahl, B., Butters, R., Vanderloo, M., & Wood, D. S. (2017). Short-Term Interventions for Survivors of Intimate Partner Violence: A Systematic Review and Meta-Analysis. *Trauma, Violence & Abuse*, *18*(2), 155–171. https://doi.org/10.1177/1524838015602736

Awolaran, O., Olubumuyi, O., OlaOlorun, F., Assink, M., van Rooij, F., & Leijten, P. (2022). Interventions to reduce intimate partner violence against women in low- and middle-income countries: A meta-analysis. *Aggression and Violent Behavior*, *64*(101746). https://doi.org/10.1016/j.avb.2022.101746

Babaee, E., Tehrani-Banihashem, A., Asadi-Aliabadi, M., Sheykholeslami, A.,

Purabdollah, M., Ashari, A., & Nojomi, M. (2021). Population-Based Approaches to Prevent Domestic Violence against Women Using a Systematic Review. *Iranian Journal of Psychiatry*, *16*(1), 94–105. https://doi.org/10.18502/ijps.v16i1.5384

Bacchus, L. J., Colombini, M., Contreras Urbina, M., Howarth, E., Gardner, F., Annan, J., Ashburn, K., Madrid, B., Levtov, R., & Watts, C. (2017). Exploring opportunities for coordinated responses to intimate partner violence and child maltreatment in low and middle income countries: A scoping review. *Psychology, Health & Medicine*, *22*(sup1), 135–165. https://doi.org/10.1080/13548506.2016.1274410

Bair-Merritt, M. H., Lewis-O'Connor, A., Goel, S., Amato, P., Ismailji, T., Jelley, M., Lenahan, P., & Cronholm, P. (2014). Primary care-based interventions for intimate partner violence: A systematic review. *American Journal of Preventive Medicine*, *46*(2), 188–194. https://doi.org/10.1016/j.amepre.2013.10.001

Baptista, R. R. de O., & Tagliamento, G. (2021). Effectiveness of interventions with male perpetrators of violence against women: A narrative review. *Aggression and Violent Behavior*, *58*, 101583. https://doi.org/10.1016/j.avb.2021.101583

Baranov, V., Cameron, L., Contreras Suarez, D., & Thibout, C. (2021). Theoretical Underpinnings and Meta-analysis of the Effects of Cash Transfers on Intimate Partner Violence in Low- and Middle-Income Countries. *The Journal of Development Studies*, *57*(1), 1–25. https://

doi.org/10.1080/00220388.2020.1762859

Barnett, G. D., & Fitzalan Howard, F. (2018). What doesn't work to reduce reoffending? A review of reviews of ineffective interventions for adults convicted of crimes. *European Psychologist*, *23*(2), 111–129. https://doi.org/10.1027/1016-9040/a000323

Beek, E. T., Spruit, A., Kuiper, C. H. Z., van der Rijken, R. E. A., Hendriks, J., & Stams, G. J. J. M. (2018). Treatment Effect on Recidivism for Juveniles Who Have Sexually Offended: A Multilevel Meta-Analysis. *Journal of Abnormal Child Psychology*, *46*(3), 543–556. https://doi.org/10.1007/s10802-017-0308-3

Bourey, C., Williams, W., Bernstein, E. E., & Stephenson, R. (2015). Systematic review of structural interventions for intimate partner violence in low- and middle-income countries: Organizing evidence for prevention. *BMC Public Health*, *15*, 1165. https://doi.org/10.1186/s12889-015-2460-4

Bowring, A. L., Wright, C. J. C., Douglass, C., Gold, J., & Lim, M. S. C. (2018). Features of successful sexual health promotion programs for young people: Findings from a review of systematic reviews. *Health Promotion Journal of Australia: Official Journal of Australian Association of Health Promotion Professionals*, *29*(1), 46–57. https://doi.org/10.1002/hpja.3

Brooks, O., Burman, M., Lombard, N., McIvor, G., Stevenson-Hastings, L., & Kyle, D. (2014). 'Violence against women: Effective interventions and practices with perpetrators: a literature review. *The Scottish Centre for Crime & Justice Research*. http://www.sccjr.ac.uk/publications/violence-

against-women-effective-interventions-and-practices-with-perpetrators/

Buller, A. M., Peterman, A., Ranganathan, M., Bleile, A., Hidrobo, M., & Heise, L. (2018). A Mixed-Method Review of Cash Transfers and Intimate Partner Violence in Low- and Middle-Income Countries. *The World Bank Research Observer*, *33*(2), 218–258. https://doi.org/10.1093/wbro/lky002

Carr, A. (2019). Couple therapy, family therapy and systemic interventions for adult-focused problems: The current evidence base. *Journal of Family Therapy*, *41*(4), 492–536. https://doi.org/10.1111/1467-6427.12225

Chen, M., & Chan, K. L. (2022). Effectiveness of Digital Health Interventions on Unintentional Injury, Violence, and Suicide: Meta-Analysis. *Trauma, Violence & Abuse*, *23*(2), 605–619. https://doi.org/10.1177/1524838020967346

Cheng, S.-Y., Davis, M., Jonson-Reid, M., & Yaeger, L. (2021). Compared to What? A Meta-Analysis of Batterer Intervention Studies Using Nontreated Controls or Comparisons. *Trauma, Violence & Abuse*, *22*(3), 496–511. https://doi.org/10.1177/1524838019865927

Choo, E. K., Gottlieb, A. S., DeLuca, M., Tape, C., Colwell, L., & Zlotnick, C. (2015). Systematic Review of ED-based Intimate Partner Violence Intervention Research. *The Western Journal of Emergency Medicine*, *16*(7), 1037–1042. https://doi.org/10.5811/westjem.2015.10.27586

Cleaver, K., Maras, P., Oram, C., & McCallum, K. (2019). A review of UK based multi-agency approaches to early intervention in domestic abuse: Lessons to be learnt from existing evaluation studies. *Aggression and Violent*

Behavior, *46*, 140–155. https://doi.org/10.1016/j.avb.2019.02.005

Cork, C., White, R., Noel, P., & Bergin, N. (2020). Randomized Controlled Trials of Interventions Addressing Intimate Partner Violence in Sub-Saharan Africa: A Systematic Review. *Trauma, Violence & Abuse*, *21*(4), 643–659. https://doi.org/10.1177/1524838018784585

Crane, C. A., & Easton, C. J. (2017). Integrated treatment options for male perpetrators of intimate partner violence. *Drug and Alcohol Review*, *36*(1), 24–33. https://doi.org/10.1111/dar.12496

Crooks, C. V., Jaffe, P., Dunlop, C., Kerry, A., & Exner-Cortens, D. (2019). Preventing Gender-Based Violence Among Adolescents and Young Adults: Lessons From 25 Years of Program Development and Evaluation. *Violence Against Women*, *25*(1), 29–55. https://doi.org/10.1177/1077801218815778

Cunha, O., & Gonçalves, R. A. (2014). The current practices of intervention with batterers. *Archives of Clinical Psychiatry (São Paulo)*, *41*, 40–48. https://doi.org/10.1590/0101-60830000000008

Daley, D., McCauley, M., & van den Broek, N. (2020). Interventions for women who report domestic violence during and after pregnancy in low- and middle-income countries: A systematic literature review. *BMC Pregnancy and Childbirth*, *20*(1), 141. https://doi.org/10.1186/s12884-020-2819-0

De Koker, P., Mathews, C., Zuch, M., Bastien, S., & Mason-Jones, A. J. (2014). A systematic review of interventions for preventing adolescent intimate partner violence. *The Journal of Adolescent Health: Official Publication of*

the Society for Adolescent Medicine, *54*(1), 3–13. https://doi.org/10.1016/j.jadohealth.2013.08.008

De La Rue, L., Polanin, J. R., Espelage, D. L., & Pigott, T. D. (2014). School-Based Interventions to Reduce Dating and Sexual Violence: A Systematic Review. *Campbell Systematic Reviews*, *10*(1), 1–110. https://doi.org/10.4073/csr.2014.7

De La Rue, L., Polanin, J. R., Espelage, D. L., & Pigott, T. D. (2017). A meta-analysis of school-based interventions aimed to prevent or reduce violence in teen dating relationships. *Review of Educational Research*, *87*(1), 7–34. https://doi.org/10.3102/0034654316632061

De Oliveira, R. N. G., Gessner, R., Brancaglioni, B. de C. A., Fonseca, R. M. G. S. da, & Egry, E. Y. (2016). Preventing violence by intimate partners in adolescence: An integrative review. *Revista Da Escola De Enfermagem Da U S P*, *50*(1), 137–147. https://doi.org/10.1590/S0080-623420160000100018

DeGue, S., Valle, L. A., Holt, M. K., Massetti, G. M., Matjasko, J. L., & Tharp, A. T. (2014). A systematic review of primary prevention strategies for sexual violence perpetration. *Aggression and Violent Behavior*, *19*(4), 346–362. https://doi.org/10.1016/j.avb.2014.05.004

Denhard, L., Mahoney, P., Kim, E., & Gielen, A. (2020). A Review of Alcohol Use Interventions on College Campuses and Sexual Assault Outcomes. *Current Epidemiology Reports*, *7*(4), 363–375. https://doi.org/10.1007/s40471-020-00253-2

Dowling, C., Morgan, A., Boyd, C., & Voce, I. (2018). Policing domestic violence: A review of the evidence. *Australian Institute of Criminology*, *13*. https://www.aic.gov.au/publications/rr/rr13

Dowling, C., Morgan, A., Hulme, S., Manning, M., & Wong, G. (2018). Protection orders for domestic violence: A systematic review. *Trends & Issues in Crime and Criminal Justice*, *551*. Canberra: Australian Institute of Criminology. https://doi.org/10.52922/ti116237

Dworkin, S. L., Treves-Kagan, S., & Lippman, S. A. (2013). Gender-transformative interventions to reduce HIV risks and violence with heterosexually-active men: A review of the global evidence. *AIDS and Behavior*, *17*(9), 2845–2863. https://doi.org/10.1007/s10461-013-0565-2

Eckhardt, C. I., Murphy, C. M., Whitaker, D. J., Sprunger, J., Dykstra, R., & Woodard, K. (2013). The effectiveness of intervention programs for perpetrators and victims of intimate partner violence. *Partner Abuse*, *4*(2), 196–231. https://doi.org/10.1891/1946-6560.4.2.196

Eggers Del Campo, I., & Steinert, J. I. (2022). The Effect of Female Economic Empowerment Interventions on the Risk of Intimate Partner Violence: A Systematic Review and Meta-Analysis. *Trauma, Violence & Abuse*, *23*(3), 810–826. https://doi.org/10.1177/1524838020976088

El Morr, C., & Layal, M. (2020). Effectiveness of ICT-based intimate partner violence interventions: A systematic review. *BMC Public Health*, *20*(1), 1372. https://doi.org/10.1186/s12889-020-09408-8

Ellsberg, M., Arango, D. J., Morton, M., Gennari, F., Kiplesund, S., Contreras, M.,

& Watts, C. (2015). Prevention of violence against women and girls: What does the evidence say? *The Lancet*, *385*(9977), 1555–1566. https://doi.org/10.1016/S0140-6736(14)61703-7

Emezue, C. N., Williams, O. J., & Bloom, T. L. (2021). Culturally-differentiated batterer intervention programs for immigrant male batterers (IMB): An integrative review. *Journal of Aggression, Maltreatment & Trauma*, *30*(7), 907–930. https://doi.org/10.1080/10926771.2019.1685042

Esquivel Santoveña, E. E., & da Silva, T. (2016). Domestic violence intervention programs for perpetrators in Latin America and the Caribbean. *Partner Abuse*, *7*(3), 316–352. https://doi.org/10.1891/1946-6560.7.3.316

Evans, J. L., Burroughs, M. E., & Knowlden, A. P. (2019). Examining the efficacy of bystander sexual violence interventions for first-year college students: A systematic review. *Aggression and Violent Behavior*, *48*, 72–82. https://doi.org/10.1016/j.avb.2019.08.016

Fagan, A. A., & Catalano, R. F. (2013). What Works in Youth Violence Prevention: A Review of the Literature. *Research on Social Work Practice*, *23*(2), 141–156. https://doi.org/10.1177/1049731512465899

Fellmeth, G. L. T., Heffernan, C., Nurse, J., Habibula, S., & Sethi, D. (2013). Educational and skills-based interventions for preventing relationship and dating violence in adolescents and young adults. *The Cochrane Database of Systematic Reviews*, *6*, CD004534. https://doi.org/10.1002/14651858.CD004534.pub3

Feltner, C., Wallace, I., Berkman, N., Kistler, C. E., Middleton, J. C., Barclay, C.,

Higginbotham, L., Green, J. T., & Jonas, D. E. (2018). Screening for Intimate Partner Violence, Elder Abuse, and Abuse of Vulnerable Adults: Evidence Report and Systematic Review for the US Preventive Services Task Force. *JAMA*, *320*(16), 1688–1701. https://doi.org/10.1001/jama.2018.13212

Fenton, R. A., Mott, H. L., McCartan, K., & Rumney, P. (2016). A review of evidence for bystander intervention to prevent sexual and domestic violence in universities. *Public Health England*. https://uwe-repository.worktribe.com/output/918734/a-review-of-evidence-for-bystander-intervention-to-prevent-sexual-and-domestic-violence-in-universities

Fernández-Fernández, R., Navas, M. P., & Sobral, J. (2022). What is Known about the Intervention with Gender Abusers? A Meta-analysis on Intervention Effectiveness. *Anuario de Psicologia Juridica*, *32*(1), 23–31. https://doi.org/10.5093/apj2021a17

Ferreira, M. N. X., Hino, P., Taminato, M., & Fernandes, H. (2019). Care of perpetrators of repeat family violence: An integrative literature review. *Acta Paul Enferm.*, *32*(3), Article 3. https://doi.org/10.1590/1982-0194201900046

Feyissa, G. T., Lockwood, C., & Munn, Z. (2015). The effectiveness of home-based HIV counseling and testing on reducing stigma and risky sexual behavior among adults and adolescents: A systematic review and meta-analyses. *JBI Database of Systematic Reviews and Implementation*

Reports, *13*(6), 318–372. https://doi.org/10.11124/jbisrir-2015-2235

Finnie, R. K. C., Okasako-Schmucker, D. L., Buchanan, L., Carty, D., Wethington, H., Mercer, S. L., Basile, K. C., DeGue, S., Niolon, P. H., Bishop, J., Titus, T., Noursi, S., Dickerson, S. A., Whitaker, D., Swider, S., Remington, P., & Community Preventive Services Task Force. (2022). Intimate Partner and Sexual Violence Prevention Among Youth: A Community Guide Systematic Review. *American Journal of Preventive Medicine*, *62*(1), e45–e55. https://doi.org/10.1016/j.amepre.2021.06.021

Fox, K. A., & Shjarback, J. A. (2016). What Works to Reduce Victimization? Synthesizing What We Know and Where to Go From Here. *Violence and Victims*, *31*(2), 285–319. https://doi.org/10.1891/0886-6708.VV-D-14-00146

Fulu, E., Kerr-Wilson, A., & Lang, J. (2014). *What works to prevent violence against women and girls? Evidence Review of interventions to prevent violence against women and girls* (assets.publishing.service.gov.uk). Gov.UK. chrome-extension://efaidnbmnnnibpcajpcglclefindmkaj/https://assets.publishing.service.gov.uk/media/57a089a8ed915d3cfd00037c/What_Works_Inception_Report_June_2014_AnnexF_WG23_paper_prevention_interventions.pdf

Gannon, T. A., Olver, M. E., Mallion, J. S., & James, M. (2019). Does specialized psychological treatment for offending reduce recidivism? A meta-analysis examining staff and program variables as predictors of treatment effectiveness. *Clinical Psychology Review*, *73*, 101752. https://doi.org/

10.1016/j.cpr.2019.101752

Garner, J., Maxwell, C., & Lee, J. (2021). The Specific Deterrent Effects of Criminal Sanctions for Intimate Partner Violence: A Meta-Analysis. *Journal of Criminal Law and Criminology*, *111*(1), 227.

Gibbs, A., Jacobson, J., & Kerr Wilson, A. (2017). A global comprehensive review of economic interventions to prevent intimate partner violence and HIV risk behaviours. *Global Health Action*, *10*(sup2), 1290427. https://doi.org/10.1080/16549716.2017.1290427

Gichuru, W., Ojha, S., Smith, S., Smyth, A. R., & Szatkowski, L. (2019). Is microfinance associated with changes in women's well-being and children's nutrition? A systematic review and meta-analysis. *BMJ Open*, *9*(1), e023658. https://doi.org/10.1136/bmjopen-2018-023658

Gilani, T. M., Simbar, M., Kariman, N., Gilani, T. M., Bazzazian, S., Ghiasvand, M., Hajiesmaello, M., & Kazemi, S. (2020). Methods for Prevention of Sexual Abuse among Adolescents: A Systematic Review. *Iranian Journal of Public Health*, *49*(6), 1060–1068. https://doi.org/10.18502/ijph.v49i6.3357

Gilbert, L., Raj, A., Hien, D., Stockman, J., Terlikbayeva, A., & Wyatt, G. (2015). Targeting the SAVA (Substance Abuse, Violence, and AIDS) Syndemic Among Women and Girls: A Global Review of Epidemiology and Integrated Interventions. *Journal of Acquired Immune Deficiency Syndromes (1999)*, *69 Suppl 2*(0 2), S118-127. https://doi.org/10.1097/QAI.0000000000000626

Gilchrist, G., Munoz, J. T., & Easton, C. J. (2015). Should we reconsider anger management when addressing physical intimate partner violence perpetration by alcohol abusing males? A systematic review. *Aggression and Violent Behavior*, *25*(Part A), 124–132. https://doi.org/10.1016/j.avb.2015.07.008

Giusto, A., & Puffer, E. (2018). A systematic review of interventions targeting men's alcohol use and family relationships in low- and middle-income countries. *Global Mental Health*, *5*, e10. https://doi.org/10.1017/gmh.2017.32

Goldfarb, E. S., & Lieberman, L. D. (2021). Three Decades of Research: The Case for Comprehensive Sex Education. *Journal of Adolescent Health*, *68*(1), 13–27. https://doi.org/10.1016/j.jadohealth.2020.07.036

Graham, L. M., Embry, V., Young, B.-R., Macy, R. J., Moracco, K. E., Reyes, H. L. M., & Martin, S. L. (2021). Evaluations of Prevention Programs for Sexual, Dating, and Intimate Partner Violence for Boys and Men: A Systematic Review. *Trauma, Violence & Abuse*, *22*(3), 439–465. https://doi.org/10.1177/1524838019851158

Hackett, S., McWhirter, P. T., & Lesher, S. (2016). The Therapeutic Efficacy of Domestic Violence Victim Interventions. *Trauma, Violence & Abuse*, *17*(2), 123–132. https://doi.org/10.1177/1524838014566720

Hameed, M., O'Doherty, L., Gilchrist, G., Tirado-Muñoz, J., Taft, A., Chondros, P., Feder, G., Tan, M., & Hegarty, K. (2020). Psychological therapies for women who experience intimate partner violence. *The Cochrane*

Database of Systematic Reviews, *7*(7), CD013017. https://doi.org/10.1002/14651858.CD013017.pub2

Hardee, K., Gay, J., Croce-Galis, M., & Peltz, A. (2014). Strengthening the enabling environment for women and girls: What is the evidence in social and structural approaches in the HIV response? *Journal of the International AIDS Society*, *17*(1), 18619. https://doi.org/10.7448/IAS.17.1.18619

Heard, E., Mutch, A., & Fitzgerald, L. (2020). Using Applied Theater in Primary, Secondary, and Tertiary Prevention of Intimate Partner Violence: A Systematic Review. *Trauma, Violence & Abuse*, *21*(1), 138–156. https://doi.org/10.1177/1524838017750157

Hollander, J. A. (2018). Women's self-defense and sexual assault resistance: The state of the field. *Sociology Compass*, *12*(8), e12597. https://doi.org/10.1111/soc4.12597

Hoppe, S. J., Zhang, Y., Hayes, B. E., & Bills, M. A. (2020). Mandatory arrest for domestic violence and repeat offending: A meta-analysis. *Aggression and Violent Behavior*, *53*, 101430. https://doi.org/10.1016/j.avb.2020.101430

Howell, K. H., Miller-Graff, L. E., Hasselle, A. J., & Scrafford, K. E. (2017). The unique needs of pregnant, violence-exposed women: A systematic review of current interventions and directions for translational research. *Aggression and Violent Behavior*, *34*, 128–138. https://doi.org/10.1016/j.avb.2017.01.021

Hudspith, L. F., Wager, N., Willmott, D., & Gallagher, B. (2023). Forty Years of

Rape Myth Acceptance Interventions: A Systematic Review of What Works in Naturalistic Institutional Settings and How this can be Applied to Educational Guidance for Jurors. *Trauma, Violence, & Abuse*, *24*(2), 981–1000. https://doi.org/10.1177/15248380211050575

Jahanfar, S., Howard, L. M., & Medley, N. (2014). Interventions for preventing or reducing domestic violence against pregnant women. *The Cochrane Database of Systematic Reviews*, *2014*(11), CD009414. https://doi.org/10.1002/14651858.CD009414.pub3

Jewkes, R., McLean Hilker, L., Khan, S., Fulu, E., Busiello, F., & Fraser, E. (2015). *What Works to Prevent Violence Against Women and Girls Evidence Reviews Paper 3: Response mechanisms to prevent violence against women and girls* (assets.publishing.service.gov.uk; pp. 1–58). UK DFID. https://resourcecentre.savethechildren.net/pdf/2015_ww_evidence_review_3.pdf/

Jewkes, R., Stern, E., & Ramsoomar, L. (2019). *Preventing violence against women and girls: Community activism approaches to shift harmful gender attitudes, roles and social norms* (www.whatworks.co.za; pp. 2–15). What Works. https://www.alignplatform.org/resources/preventing-violence-against-women-and-girls-community-activism-approaches-shift-harmful

Jonker, I. E., Sijbrandij, M., van Luijtelaar, M. J. A., Cuijpers, P., & Wolf, J. R. L. M. (2015). The effectiveness of interventions during and after residence in women's shelters: A meta-analysis. *European Journal of Public Health*, *25*(1), 15–19. https://doi.org/10.1093/eurpub/cku092

Jouriles, E. N., Krauss, A., Vu, N. L., Banyard, V. L., & McDonald, R. (2018). Bystander programs addressing sexual violence on college campuses: A systematic review and meta-analysis of program outcomes and delivery methods. *Journal of American College Health: J of ACH*, *66*(6), 457–466. https://doi.org/10.1080/07448481.2018.1431906

Karakurt, G., Koç, E., Çetinsaya, E. E., Ayluçtarhan, Z., & Bolen, S. (2019). Meta-analysis and systematic review for the treatment of perpetrators of intimate partner violence. *Neuroscience and Biobehavioral Reviews*, *105*, 220–230. https://doi.org/10.1016/j.neubiorev.2019.08.006

Karakurt, G., Whiting, K., Van Esch, C., Bolen, S., & Calabrese, J. (2016). Couple Therapy for Intimate Partner Violence: A Systematic Review and Meta-Analysis. *Journal of Marital and Family Therapy*, *42*(4), 567–583. https://doi.org/10.1111/jmft.12178

Katz, J., & Moore, J. (2013). Bystander education training for campus sexual assault prevention: An initial meta-analysis. *Violence and Victims*, *28*(6), 1054–1067. https://doi.org/10.1891/0886-6708.vv-d-12-00113

Keith, T., Hyslop, F., & Richmond, R. (2022). A Systematic Review of Interventions to Reduce Gender-Based Violence Among Women and Girls in Sub-Saharan Africa. *Trauma, Violence & Abuse*, *24*(3), 1443–1464. https://doi.org/10.1177/15248380211068136

Kennedy, C. E., Fonner, V. A., O'Reilly, K. R., & Sweat, M. D. (2014). A systematic review of income generation interventions, including microfinance and vocational skills training, for HIV prevention. *AIDS Care*,

26(6), 659–673. https://doi.org/10.1080/09540121.2013.845287

Kerr Wilson, A., Gibbs, A., McAslan Fraser, E., Ramsoomar, L., Parke, A., M A Khuwaja, H., & Jewkes, R. (2020). *What Works—A rigorous global evidence review of interventions to prevent violence against women and girls, What Works to prevent violence against women and girls global Programme, Pretoria, South Africa* (https://www.whatworks.co.za/; What Works to Prevent Violence Against Women and Girls Global Programme, Pretoria, South Africa). What Works: Evidence Hub, What Works Resources. https://www.whatworks.co.za/resources/item/693-a-rigorous-global-evidence-review-of-interventions-to-prevent-violence-against-women-and-girls

Kettrey, H. H., & Marx, R. A. (2019a). Does the Gendered Approach of Bystander Programs Matter in the Prevention of Sexual Assault Among Adolescents and College Students? A Systematic Review and Meta-Analysis. *Archives of Sexual Behavior*, *48*(7), 2037–2053. https://doi.org/10.1007/s10508-019-01503-1

Kettrey, H. H., & Marx, R. A. (2019b). The Effects of Bystander Programs on the Prevention of Sexual Assault across the College Years: A Systematic Review and Meta-analysis. *Journal of Youth and Adolescence*, *48*(2), 212–227. https://doi.org/10.1007/s10964-018-0927-1

Kettrey, H. H., & Marx, R. A. (2021). Effects of bystander sexual assault prevention programs on promoting intervention skills and combatting the bystander effect: A systematic review and meta-analysis. *Journal of*

Experimental Criminology, *17*(3), 343–367. https://doi.org/10.1007/s11292-020-09417-y

Kiani, Z., Simbar, M., Fakari, F. R., Kazemi, S., Ghasemi, V., Azimi, N., Mokhtariyan, T., & Bazzazian, S. (2021). A systematic review: Empowerment interventions to reduce domestic violence? *Aggression and Violent Behavior*, *58*. https://doi.org/10.1016/j.avb.2021.101585

Kim, B., Benekos, P. J., & Merlo, A. V. (2016). Sex Offender Recidivism Revisited: Review of Recent Meta-analyses on the Effects of Sex Offender Treatment. *Trauma, Violence & Abuse*, *17*(1), 105–117. https://doi.org/10.1177/1524838014566719

Kirk, L., Terry, S., Lokuge, K., & Watterson, J. L. (2017). Effectiveness of secondary and tertiary prevention for violence against women in low and low-middle income countries: A systematic review. *BMC Public Health*, *17*(1), 622. https://doi.org/10.1186/s12889-017-4502-6

Kirk-Provencher, K. T., Spillane, N. S., Schick, M. R., Chalmers, S. J., Hawes, C., & Orchowski, L. M. (2023). Sexual and Gender Minority Inclusivity in Bystander Intervention Programs to Prevent Violence on College Campuses: A Critical Review. *Trauma, Violence, & Abuse*, *24*(1), 110–124. https://doi.org/10.1177/15248380211021606

Klein, L. B., Chesworth, B. R., Howland-Myers, J. R., Rizo, C. F., & Macy, R. J. (2021). Housing Interventions for Intimate Partner Violence Survivors: A Systematic Review. *Trauma, Violence & Abuse*, *22*(2), 249–264. https://doi.org/10.1177/1524838019836284

Långström, N., Enebrink, P., Laurén, E.-M., Lindblom, J., Werkö, S., & Hanson, R. K. (2013). Preventing sexual abusers of children from reoffending: Systematic review of medical and psychological interventions. *BMJ*, *347*, f4630. https://doi.org/10.1136/bmj.f4630

Leigh, S., & Davies, J. (2022). A rapid evidence assessment of psychological treatment approaches for stalking behaviour. *The Journal of Forensic Practice*, *24*(1), 48–62. https://doi.org/10.1108/JFP-06-2021-0039

Leite, T. H., Moraes, C. L. de, Marques, E. S., Caetano, R., Braga, J. U., & Reichenheim, M. E. (2019). Women economic empowerment via cash transfer and microcredit programs is enough to decrease intimate partner violence? Evidence from a systematic review. *Cadernos De Saude Publica*, *35*(9), e00174818. https://doi.org/10.1590/0102-311X00174818

Lester, S., Lawrence, C., & Ward, C. L. (2017). What do we know about preventing school violence? A systematic review of systematic reviews. *Psychology, Health & Medicine*, *22*(sup1), 187–223. https://doi.org/10.1080/13548506.2017.1282616

Lilley-Walker, S.-J., Hester, M., & Turner, W. (2018). Evaluation of European Domestic Violence Perpetrator Programmes: Toward a Model for Designing and Reporting Evaluations Related to Perpetrator Treatment Interventions. *International Journal of Offender Therapy and Comparative Criminology*, *62*(4), 868–884. https://doi.org/10.1177/0306624X16673853

Linde, D. S., Bakiewicz, A., Normann, A. K., Hansen, N. B., Lundh, A., & Rasch, V. (2020). Intimate Partner Violence and Electronic Health Interventions:

Systematic Review and Meta-Analysis of Randomized Trials. *Journal of Medical Internet Research*, *22*(12), e22361. 12/14/2023. https://doi.org/10.2196/22361

Lorenzetti, L. M. J., Leatherman, S., & Flax, V. L. (2017). Evaluating the effect of integrated microfinance and health interventions: An updated review of the evidence. *Health Policy and Planning*, *32*(5), 732–756. https://doi.org/10.1093/heapol/czw170

Lundgren, R., & Amin, A. (2015). Addressing intimate partner violence and sexual violence among adolescents: Emerging evidence of effectiveness. *The Journal of Adolescent Health: Official Publication of the Society for Adolescent Medicine*, *56*(1 Suppl), S42-50. https://doi.org/10.1016/j.jadohealth.2014.08.012

Malhotra, K., Gonzalez-Guarda, R. M., & Mitchell, E. M. (2015). A Review of Teen Dating Violence Prevention Research: What About Hispanic Youth? *Trauma, Violence & Abuse*, *16*(4), 444–465. https://doi.org/10.1177/1524838014537903

Marcus, R., Rivett, J., & Kruja, K. (2021). How far do parenting programmes help change norms underpinning violence against adolescents? Evidence from low and middle-income countries. *Global Public Health*, *16*(6), 820–841. https://doi.org/10.1080/17441692.2020.1776364

Marshall, K. J., Fowler, D. N., Walters, M. L., & Doreson, A. B. (2018). Interventions that Address Intimate Partner Violence and HIV Among Women: A Systematic Review. *AIDS and Behavior*, *22*(10), 3244–3263.

https://doi.org/10.1007/s10461-017-2020-2

Martínez Fortún López, M. I., Gesteira Santos, C., Morán Rodríguez, N., García Vera, M. P., & Sanz Fernández, J. (2021). Programas de prevención del abuso sexual en personas con discapacidad intelectual y del desarrollo. *Revista Española de Discapacidad (REDIS)*, *9*(1), 75–100.

Matjasko, J. L., D'Inverno, A. S., Marshall, K. J., & Kearns, M. C. (2020). Microfinance and violence prevention: A review of the evidence and adaptations for implementation in the U.S. *Preventive Medicine*, *133*, 106017. https://doi.org/10.1016/j.ypmed.2020.106017

McCloskey, L. A., Boonzaier, F., Steinbrenner, S. Y., & Hunter, T. (2016). Determinants of intimate partner violence in sub-Saharan Africa: A review of prevention and intervention programs. *Partner Abuse*, *7*(3), 277–315. https://doi.org/10.1891/1946-6560.7.3.277

McNaughton Reyes, H. L., Graham, L. M., Chen, M. S., Baron, D., Gibbs, A., Groves, A. K., Kajula, L., Bowler, S., & Maman, S. (2021). Adolescent dating violence prevention programmes: A global systematic review of evaluation studies. *The Lancet. Child & Adolescent Health*, *5*(3), 223–232. https://doi.org/10.1016/S2352-4642(20)30276-5

Meinck, F., Pantelic, M., Spreckelsen, T. F., Orza, L., Little, M. T., Nittas, V., Picker, V., Bustamam, A. A., Herrero Romero, R., Diaz Mella, E. P., & Stöckl, H. (2019). Interventions to reduce gender-based violence among young people living with or affected by HIV/AIDS in low-income and middle-income countries. *AIDS (London, England)*, *33*(14), 2219–2236.

https://doi.org/10.1097/QAD.0000000000002337

Mendelson, T., & Letourneau, E. J. (2015). Parent-focused prevention of child sexual abuse. *Prevention Science*, *16*(6), 844–852. https://doi.org/10.1007/s11121-015-0553-z

Mujal, G. N., Taylor, M. E., Fry, J. L., Gochez-Kerr, T. H., & Weaver, N. L. (2021). A Systematic Review of Bystander Interventions for the Prevention of Sexual Violence. *Trauma, Violence & Abuse*, *22*(2), 381–396. https://doi.org/10.1177/1524838019849587

Nesset, M. B., Lara-Cabrera, M. L., Dalsbø, T. K., Pedersen, S. A., Bjørngaard, J. H., & Palmstierna, T. (2019). Cognitive behavioural group therapy for male perpetrators of intimate partner violence: A systematic review. *BMC Psychiatry*, *19*(1), 11. https://doi.org/10.1186/s12888-019-2010-1

Neville, F. G., Goodall, C. A., Williams, D. J., & Donnelly, P. D. (2014). Violence brief interventions: A rapid review. *Aggression and Violent Behavior*, *19*(6), 692–698. https://doi.org/10.1016/j.avb.2014.09.015

Newlands, R. (2016). A Critical Review of Sexual Violence Prevention on College Campuses. *Acta Psychopathologica*, *02*. https://doi.org/10.4172/2469-6676.100040

NICE. (2013). Review of Interventions to Identify, Prevent, Reduce and Respond to Domestic Violence. *British Columbia Centre of Excellence for Women's Health*. National Institute for Healthcare and Excellence.

Nikolova, S. P., & Small, E. (2018). Review of the evidence of gender-focused interventions including men to reduce HIV risk and violence against

women in sub-Saharan Africa. *Journal of HIV/AIDS & Social Services*, *17*(2), 87–117. https://doi.org/10.1080/15381501.2017.1407727

Niland, K., Zukiewicz, M., & Sama-Miller, E. (2020). *What the Evidence Says: Intimate Partner Violence and Home Visiting* (19). Office of Planning, Research, and Evaluation, Administration for Children and Families, U.S Department of Health and Human Services. https://www.acf.hhs.gov/opre/report/what-evidence-says-intimate-partner-violence-and-home-visiting

O'Connor, A., Morris, H., Panayiotidis, A., Cooke, V., & Skouteris, H. (2021). Rapid Review of Men's Behavior Change Programs. *Trauma, Violence, & Abuse*, *22*(5), 1068–1085. https://doi.org/10.1177/1524838020906527

O'Doherty, L., Hegarty, K., Ramsay, J., Davidson, L. L., Feder, G., & Taft, A. (2015). Screening women for intimate partner violence in healthcare settings. *The Cochrane Database of Systematic Reviews*, *2015*(7), CD007007. https://doi.org/10.1002/14651858.CD007007.pub3

Ogunjimi, A. I., Oliveira, W. A. de, Vasconcelos, E. M. R. de, & Silva, M. A. I. (2017). Child sexual abuse prevention: Integrative review. *Journal of Nursing UFPE On Line*, *11*(11), 4469–4482.

O'Malley, T. L., & Burke, J. G. (2017). A systematic review of microfinance and women's health literature: Directions for future research. *Global Public Health*, *12*(11), 1433–1460. https://doi.org/10.1080/17441692.2016.1170181

Orchowski, L. M., Berry-Cabán, C. S., Prisock, K., Borsari, B., & Kazemi, D. M. (2018). Evaluations of Sexual Assault Prevention Programs in Military

Settings: A Synthesis of the Research Literature. *Military Medicine*, *183*(suppl_1), 421–428. https://doi.org/10.1093/milmed/usx212

Orton, L., Pennington, A., Nayak, S., Sowden, A., White, M., & Whitehead, M. (2016). Group-based microfinance for collective empowerment: A systematic review of health impacts. *Bulletin of the World Health Organization*, *94*(9), 694-704A. https://doi.org/10.2471/BLT.15.168252

Parkes, J. L. N., Heslop, J., Johnson Ross, F., Westerveld, R., & Unterhalter, E. (2016). A Rigorous Review of Global Research Evidence on Policy and Practice on School-Related Gender-Based Violence. In *UNICEF: New York, USA.* [Report]. UNICEF. https://www.unicef.org/education/files/SRGBV_review_FINAL_V1_web_version.pdf

Pérez-Martínez, V., Marcos-Marcos, J., Cerdán-Torregrosa, A., Briones-Vozmediano, E., Sanz-Barbero, B., Davó-Blanes, Mc., Daoud, N., Edwards, C., Salazar, M., La Parra-Casado, D., & Vives-Cases, C. (2023). Positive Masculinities and Gender-Based Violence Educational Interventions Among Young People: A Systematic Review. *Trauma, Violence & Abuse*, *24*(2), 468–486. https://doi.org/10.1177/15248380211030242

Petering, R., Wenzel, S., & Winetrobe, H. (2014). Systematic Review of Current Intimate Partner Violence Prevention Programs and Applicability to Homeless Youth. *Journal of the Society for Social Work and Research*, *5*(1), 107–135. https://doi.org/10.1086/675851

Piolanti, A., & Foran, H. M. (2022). Efficacy of Interventions to Prevent Physical

and Sexual Dating Violence Among Adolescents: A Systematic Review and Meta-analysis. *JAMA Pediatrics*, *176*(2), 142–149. https://doi.org/10.1001/jamapediatrics.2021.4829

Prosman, G.-J., Lo Fo Wong, S. H., van der Wouden, J. C., & Lagro-Janssen, A. L. M. (2015). Effectiveness of home visiting in reducing partner violence for families experiencing abuse: A systematic review. *Family Practice*, *32*(3), 247–256. https://doi.org/10.1093/fampra/cmu091

Ramsoomar, L., Gibbs, A., Machisa, M., Chirwa, E., Kane, J., & Jewkes, R. (2019). *Associations between Alcohol, Poor Mental Health and Intimate Partner Violence*. UK AID; What Works: Evidence Hub, What Works Resources. https://www.whatworks.co.za/resources/evidence-reviews/item/680-associations-between-alcohol-poor-mental-health-and-intimate-partner-violence

Righi, M. K., Orchowski, L. M., & Kuo, C. (2019). Integrated Intimate Partner Violence and Human Immunodeficiency Virus Interventions in Sub-Saharan Africa: A Systematic Review Targeting or Including Adolescents. *Violence and Gender*, *6*(2), 92–104. https://doi.org/10.1089/vio.2018.0027

Rivas, C., Ramsay, J., Sadowski, L., Davidson, L. L., Dunne, D., Eldridge, S., Hegarty, K., Taft, A., & Feder, G. (2015). Advocacy interventions to reduce or eliminate violence and promote the physical and psychosocial well-being of women who experience intimate partner abuse. *The Cochrane Database of Systematic Reviews*, *2015*(12), CD005043. https://doi.org/10.1002/14651858.CD005043.pub3

Rivas, C., Vigurs, C., Cameron, J., & Yeo, L. (2019). A realist review of which advocacy interventions work for which abused women under what circumstances. *Cochrane Database of Systematic Reviews*, *6*. https://doi.org/10.1002/14651858.CD013135.pub2

Robbers, G. M. L., & Morgan, A. (2017). Programme potential for the prevention of and response to sexual violence among female refugees: A literature review. *Reproductive Health Matters*, *25*(51), 69–89. https://doi.org/10.1080/09688080.2017.1401893

Rose-Clarke, K., Bentley, A., Marston, C., & Prost, A. (2019). Peer-facilitated community-based interventions for adolescent health in low- and middle-income countries: A systematic review. *PloS One*, *14*(1), e0210468. https://doi.org/10.1371/journal.pone.0210468

Russell, K. N., Voith, L. A., & Lee, H. (2021). Randomized controlled trials evaluating adolescent dating violence prevention programs with an outcome of reduced perpetration and/or victimization: A meta-analysis. *Journal of Adolescence*, *87*, 6–14. https://doi.org/10.1016/j.adolescence.2020.12.009

Sabri, B., & Gielen, A. (2019). Integrated Multicomponent Interventions for Safety and Health Risks Among Black Female Survivors of Violence: A Systematic Review. *Trauma, Violence & Abuse*, *20*(5), 720–731. https://doi.org/10.1177/1524838017730647

Sabri, B., Greene, C., & Lucas, G. M. (2019). A systematic review of comprehensive interventions for substance abuse: Focus on victimization.

Aggression and Violent Behavior, *48*, 46–59. https://doi.org/10.1016/j.avb.2019.08.006

Salas Cubillos, N., Charry, V. C. G., Losada, L. V. Z., & Usme, O. S. D. (2020). Intervenciones en violencia de género en pareja: Articulo de Revisión de la Literatura. *Revista Cuidarte*, *11*(3), Article 3. https://doi.org/10.15649/cuidarte.980

Santirso, F. A., Gilchrist, G., Lila, M., & Gracia, E. (2020). Motivational Strategies in Interventions for Intimate Partner Violence Offenders: A Systematic Review and Meta-analysis of Randomized Controlled Trials. *Psychosocial Intervention*, *29*(3), 175–190. https://doi.org/10.5093/pi2020a13

Sapkota, D., Baird, K., Saito, A., & Anderson, D. (2019). Interventions for reducing and/or controlling domestic violence among pregnant women in low- and middle-income countries: A systematic review. *Systematic Reviews*, *8*(1), 79. https://doi.org/10.1186/s13643-019-0998-4

Schmucker, M., & Lösel, F. (2015). The effects of sexual offender treatment on recidivism: An international meta-analysis of sound quality evaluations. *Journal of Experimental Criminology*, *11*(4), 597–630. https://doi.org/10.1007/s11292-015-9241-z

Semahegn, A., Torpey, K., Manu, A., Assefa, N., Tesfaye, G., & Ankomah, A. (2019). Are interventions focused on gender-norms effective in preventing domestic violence against women in low and lower-middle income countries? A systematic review and meta-analysis. *Reproductive Health*, *16*(1), 93. https://doi.org/10.1186/s12978-019-0726-5

Shorey, R. C., Tirone, V., & Stuart, G. L. (2014). Coordinated Community Response Components for Victims of Intimate Partner Violence: A Review of the Literature. *Aggression and Violent Behavior*, *19*(4), 363–371. https://doi.org/10.1016/j.avb.2014.06.001

Singh, N. S., Smith, J., Aryasinghe, S., Khosla, R., Say, L., & Blanchet, K. (2018). Evaluating the effectiveness of sexual and reproductive health services during humanitarian crises: A systematic review. *PloS One*, *13*(7), e0199300. https://doi.org/10.1371/journal.pone.0199300

Sinnott, T., & Artz, S. (2016). A LITERATURE REVIEW OF STRATEGIES FOR THE PREVENTION OF INTIMATE PARTNER VIOLENCE DURING THE CHILDBEARING YEARS. *International Journal of Child, Youth and Family Studies*, *7*(3–4), Article 3–4. https://doi.org/10.18357/ijcyfs73-4201616088

Small, E., Nikolova, S. P., & Narendorf, S. C. (2013). Synthesizing gender based HIV interventions in Sub-Sahara Africa: A systematic review of the evidence. *AIDS and Behavior*, *17*(9), 2831–2844. https://doi.org/10.1007/s10461-013-0541-x

Spangaro, J., & Ruane, J. (2014). *Health Interventions for Family and Domestic Violence: A Literature Review* (https://www.health.nsw.gov.au/; pp. 1–85). School of Social Sciences, University of New South Wales; New South Wales Health.

Spangaro, J., Toole-Anstey, C., MacPhail, C. L., Rambaldini-Gooding, D. C., Keevers, L., & Garcia-Moreno, C. (2021). The impact of interventions to reduce risk and incidence of intimate partner violence and sexual violence

in conflict and post-conflict states and other humanitarian crises in low and middle income countries: A systematic review. *Conflict and Health*, *15*(1), 86. https://doi.org/10.1186/s13031-021-00417-x

Spencer, C. M., Stith, S. M., & King, E. L. (2021). Preventing Maltreatment at Home: A Meta-Analysis Examining Outcomes From Online Programs. *Research on Social Work Practice*, *31*(2), 138–146. https://doi.org/10.1177/1049731520969978

Sprague, S., Scott, T., Garibaldi, A., Bzovsky, S., Slobogean, G. P., McKay, P., Spurr, H., Arseneau, E., Memon, M., Bhandari, M., & Swaminathan, A. (2017). A scoping review of intimate partner violence assistance programmes within health care settings. *European Journal of Psychotraumatology*, *8*(1), 1314159. https://doi.org/10.1080/20008198.2017.1314159

Stanley, N., Ellis, J., Farrelly, N., Hollinghurst, S., & Downe, S. (2015). Preventing domestic abuse for children and young people: A review of school-based interventions. *Children and Youth Services Review*, *59*, 120–131. https://doi.org/10.1016/j.childyouth.2015.10.018

Stark, L., Robinson, M. V., Seff, I., Gillespie, A., Colarelli, J., & Landis, D. (2021). The Effectiveness of Women and Girls Safe Spaces: A Systematic Review of Evidence to Address Violence Against Women and Girls in Humanitarian Contexts. *Trauma, Violence & Abuse*, *23*(4), 1249–1261. https://doi.org/10.1177/1524838021991306

Stephens-Lewis, D., Johnson, A., Huntley, A., Gilchrist, E., McMurran, M.,

Henderson, J., Feder, G., Howard, L. M., & Gilchrist, G. (2021). Interventions to Reduce Intimate Partner Violence Perpetration by Men Who Use Substances: A Systematic Review and Meta-Analysis of Efficacy. *Trauma, Violence & Abuse*, *22*(5), 1262–1278. https://doi.org/10.1177/1524838019882357

Stewart, R., Wright, B., Smith, L., Roberts, S., & Russell, N. (2021). Gendered stereotypes and norms: A systematic review of interventions designed to shift attitudes and behaviour. *Heliyon*, *7*(4), e06660. https://doi.org/10.1016/j.heliyon.2021.e06660

Stith, S. M., Topham, G. L., Spencer, C., Jones, B., Coburn, K., Kelly, L., & Langston, Z. (2022). Using systemic interventions to reduce intimate partner violence or child maltreatment: A systematic review of publications between 2010 and 2019. *Journal of Marital and Family Therapy*, *48*(1), 231–250. https://doi.org/10.1111/jmft.12566

Storer, H. L., Casey, E., & Herrenkohl, T. (2016). Efficacy of Bystander Programs to Prevent Dating Abuse Among Youth and Young Adults: A Review of the Literature. *Trauma, Violence & Abuse*, *17*(3), 256–269. https://doi.org/10.1177/1524838015584361

Tarzia, L., Forsdike, K., Feder, G., & Hegarty, K. (2020). Interventions in Health Settings for Male Perpetrators or Victims of Intimate Partner Violence. *Trauma, Violence & Abuse*, *21*(1), 123–137. https://doi.org/10.1177/1524838017744772

Tirado-Muñoz, J., Gilchrist, G., Farré, M., Hegarty, K., & Torrens, M. (2014). The

efficacy of cognitive behavioural therapy and advocacy interventions for women who have experienced intimate partner violence: A systematic review and meta-analysis. *Annals of Medicine*, *46*(8), 567–586. https://doi.org/10.3109/07853890.2014.941918

Tol, W. A., Murray, S. M., Lund, C., Bolton, P., Murray, L. K., Davies, T., Haushofer, J., Orkin, K., Witte, M., Salama, L., Patel, V., Thornicroft, G., & Bass, J. K. (2019). Can mental health treatments help prevent or reduce intimate partner violence in low- and middle-income countries? A systematic review. *BMC Women's Health*, *19*(1), 34. 12/14/2023. https://doi.org/10.1186/s12905-019-0728-z

Trabold, N., McMahon, J., Alsobrooks, S., Whitney, S., & Mittal, M. (2020). A Systematic Review of Intimate Partner Violence Interventions: State of the Field and Implications for Practitioners. *Trauma, Violence & Abuse*, *21*(2), 311–325. https://doi.org/10.1177/1524838018767934

Travers, Á., McDonagh, T., Cunningham, T., Armour, C., & Hansen, M. (2021). The effectiveness of interventions to prevent recidivism in perpetrators of intimate partner violence: A systematic review and meta-analysis. *Clinical Psychology Review*, *84*, 101974. https://doi.org/10.1016/j.cpr.2021.101974

Turner, D. T., Riedel, E., Kobeissi, L. H., Karyotaki, E., Garcia-Moreno, C., Say, L., & Cuijpers, P. (2020). Psychosocial interventions for intimate partner violence in low and middle income countries: A meta-analysis of randomised controlled trials. *Journal of Global Health*, *10*(1), 010409. https://doi.org/10.7189/jogh.10.010409

van Daalen, K. R., Dada, S., James, R., Ashworth, H. C., Khorsand, P., Lim, J., Mooney, C., Khankan, Y., Essar, M. Y., Kuhn, I., Juillard, H., & Blanchet, K. (2022). Impact of conditional and unconditional cash transfers on health outcomes and use of health services in humanitarian settings: A mixed-methods systematic review. *BMJ Global Health*, *7*(1), e007902. https://doi.org/10.1136/bmjgh-2021-007902

Van Parys, A.-S., Verhamme, A., Temmerman, M., & Verstraelen, H. (2014). Intimate Partner Violence and Pregnancy: A Systematic Review of Interventions. *PLoS ONE*, *9*(1), e85084. https://doi.org/10.1371/journal.pone.0085084

Vargas, S. E., Norris, C., Landoll, R. R., Crone, B., Clark, M. F., Quinlan, J. D., & Guthrie, K. M. (2020). Interventions to Improve Sexual and Reproductive Health in US Active Duty Military Service Members: A Systematic Review. *American Journal of Health Promotion: AJHP*, *34*(5), 538–548. https://doi.org/10.1177/0890117120908511

Walsh, K., Zwi, K., Woolfenden, S., & Shlonsky, A. (2015). School-based education programmes for the prevention of child sexual abuse. *The Cochrane Database of Systematic Reviews*, *2015*(4), CD004380. https://doi.org/10.1002/14651858.CD004380.pub3

Wigham, S., McGovern, R., Kaner, E., & Hackett, S. S. (2022). A review of recent innovation in psychosocial interventions for reducing violence and aggression in adults using a horizon scanning approach. *Aggression and Violent Behavior*, *62*, 101685. https://doi.org/10.1016/j.avb.2021.101685

Williams, A. M., Heise, L., & Tas, E. O. (2022). *How Well do Economic Empowerment Efforts Prevent Intimate Partner Violence in South Asia? (English)* (1). World Bank: South Asia Region Gender Innovation Lab. https://policycommons.net/artifacts/2484312/how-well-do-economic-empowerment-efforts-prevent-intimate-partner-violence-in-south-asia-english/3506710/

Wilson, D. B., Feder, L., & Olaghere, A. (2021). Court-mandated interventions for individuals convicted of domestic violence: An updated Campbell systematic review. *Campbell Systematic Reviews*, *17*(1), e1151. https://doi.org/10.1002/cl2.1151

Wilson, I. M., Graham, K., & Taft, A. (2014). Alcohol interventions, alcohol policy and intimate partner violence: A systematic review. *BMC Public Health*, *14*(1), 881. https://doi.org/10.1186/1471-2458-14-881

Wong, J. S., Bouchard, J., & Lee, C. (2023). The Effectiveness of College Dating Violence Prevention Programs: A Meta-Analysis. *Trauma, Violence & Abuse*, *24*(2), 684–701. https://doi.org/10.1177/15248380211036058

Wright, L. A., Zounlome, N. O. O., & Whiston, S. C. (2020). The Effectiveness of Male-Targeted Sexual Assault Prevention Programs: A Meta-Analysis. *Trauma, Violence & Abuse*, *21*(5), 859–869. https://doi.org/10.1177/1524838018801330

Yanez Peñuñuri, L. Y. (2019). Therapeutic intervention for victims and perpetrators of dating violence: A systematic review. *Revista Iberoamericana de Psicología y Salud*. https://doi.org/10.23923/

j.rips.2019.02.029

Yount, K. M., Krause, K. H., & Miedema, S. S. (2017). Preventing gender-based violence victimization in adolescent girls in lower-income countries: Systematic review of reviews. *Social Science & Medicine (1982)*, *192*, 1–13. https://doi.org/10.1016/j.socscimed.2017.08.038

Zhang, H., Shi, R., Li, Y., & Wang, Y. (2021). Effectiveness of School-Based Child Sexual Abuse Prevention Programs in China: A Meta-Analysis. *Research on Social Work Practice*, *31*(7), 693–705. https://doi.org/10.1177/10497315211022827
